# Mutations in Protein-Binding Hot-Spots on the Hub Protein Smad3 Differentially Affect Its Protein Interactions and Smad3-Regulated Gene Expression

**DOI:** 10.1371/journal.pone.0025021

**Published:** 2011-09-19

**Authors:** Michelle M. Schiro, Sara E. Stauber, Tami L. Peterson, Chateen Krueger, Steven J. Darnell, Kenneth A. Satyshur, Norman R. Drinkwater, Michael A. Newton, F. Michael Hoffmann

**Affiliations:** 1 McArdle Laboratory for Cancer Research, Department of Oncology, University of Wisconsin Carbone Cancer Center, School of Medicine and Public Health, University of Wisconsin, Madison, Wisconsin, United States of America; 2 Department of Medical Genetics, School of Medicine and Public Health, University of Wisconsin, Madison, Wisconsin, United States of America; 3 Departments of Statistics and of Biostatistics and Medical Informatics, University of Wisconsin Carbone Cancer Center, School of Medicine and Public Health, University of Wisconsin, Madison, Wisconsin, United States of America; Roswell Park Cancer Institute, United States of America

## Abstract

**Background:**

Hub proteins are connected through binding interactions to many other proteins. Smad3, a mediator of signal transduction induced by transforming growth factor beta (TGF-β), serves as a hub protein for over 50 protein-protein interactions. Different cellular responses mediated by Smad3 are the product of cell-type and context dependent Smad3-nucleated protein complexes acting in concert. Our hypothesis is that perturbation of this spectrum of protein complexes by mutation of single protein-binding hot-spots on Smad3 will have distinct consequences on Smad3-mediated responses.

**Methodology/Principal Findings:**

We mutated 28 amino acids on the surface of the Smad3 MH2 domain and identified 22 Smad3 variants with reduced binding to subsets of 17 Smad3-binding proteins including Smad4, SARA, Ski, Smurf2 and SIP1. Mutations defective in binding to Smad4, e.g., D408H, or defective in nucleocytoplasmic shuttling, e.g., W406A, were compromised in modulating the expression levels of a Smad3-dependent reporter gene or six endogenous Smad3-responsive genes: Mmp9, IL11, Tnfaip6, Fermt1, Olfm2 and Wnt11. However, the Smad3 mutants Y226A, Y297A, W326A, K341A, and E267A had distinct differences on TGF-β signaling. For example, K341A and Y226A both reduced the Smad3-mediated activation of the reporter gene by ∼50% but K341A only reduced the TGF-β inducibilty of Olfm2 in contrast to Y226A which reduced the TGF-β inducibility of all six endogenous genes as severely as the W406A mutation. E267A had increased protein binding but reduced TGF-β inducibility because it caused higher basal levels of expression. Y297A had increased TGF-β inducibility because it caused lower Smad3-induced basal levels of gene expression.

**Conclusions/Significance:**

Mutations in protein binding hot-spots on Smad3 reduced the binding to different subsets of interacting proteins and caused a range of quantitative changes in the expression of genes induced by Smad3. This approach should be useful for unraveling which Smad3 protein complexes are critical for specific biological responses.

## Introduction

Transient protein-protein interactions are a ubiquitous molecular mechanism for regulating virtually every cellular function and for crosstalk between signaling pathways [1], [2]. Protein interaction mapping has identified certain proteins, called hub proteins, that are connected through binding interactions to many other proteins [3], [4], [5]. Dissecting the functional importance of the many different and potentially overlapping binding sites on a hub protein is a challenge. For example, Smad3 is a key mediator of the signal transduction processes induced by transforming growth factor beta (TGF-β) that serves as a hub protein for many different protein-binding partners [6]. TGF-β plays a prominent role in regulating a variety of cellular functions including cell migration, cell proliferation, apoptosis, differentiation, immunosuppression, inflammation, tumor-suppression, and angiogenesis [7], [8]. Elevated TGF-β signaling also has been associated with several disease states including metastasis and immune evasion by cancer cells [9], [10] and fibrosis in many tissues including skin, lung and kidney [11], [12], [13]. The specific cellular responses to TGF-β are context dependent and vary according to the cell type, the cellular environment, and the activity of other signaling pathways [14]. The broad diversity of cellular responses to TGF-β is generated primarily through two mechanisms: protein-protein interactions between the TGF-β-activated Smad2, Smad3 and Smad4 proteins and over 50 transcriptional activator and repressor proteins, and parallel activation by TGF-β of several Smad-independent signaling pathways including the Ras-MAPK pathway and the RhoA/ROCK/cofilin pathway [15], [16], [17].

Smad2 and Smad3, the receptor-regulated or R-Smads, are phosphorylated in response to TGF-β binding to its Type II and Type I receptor serine kinases [8], [18], [19]. The phosphorylated Smad2 or Smad3 MH2 domains can assemble into homomeric trimers but the preferred structure is a heterotrimer consisting of two R-Smads and one Smad4 [20], [21], [22], [23]. Although very similar in structure and primary amino acid sequence, Smad2 and Smad3 affect distinct cellular genes [24], [25], [26]. For example, Smad3 has been shown to mediate epithelial to mesenchymal transition induced by TGF-β in mouse mammary epithelial cells including changes in cell morphology, cell-cell junctions, increased MMP9 expression, reduced proliferation and increased apoptosis [27]. Smad3 also mediated the invasive behavior of a malignant subclone of MCF10A1 human breast cancer cells, in part through induction of MMP2 and MMP9 [28]. Recently, Smad3 mutations have been associated with an inherited autosomal dominant aortic aneurysm syndrome [29]. The specific involvement of Smad3 in several disease-related phenotypes regulated by TGF-β led us to focus on perturbing Smad3 protein-protein interactions in order to better understand its molecular mechanisms of action.

Smad3 has a similar structure to other Smad proteins including an N-terminal globular domain with DNA binding activity (the MH1 domain), a central linker region with regulatory sites, and a C-terminal globular domain (the MH2 domain) with transcriptional regulatory activity [30]. The MH2 domains have a central β sandwich, with a conserved three-helix bundle (H3, H4, and H5) on one end and a conserved loop-helix region (L1, L2, L3, and H1) on the other end [21]; these two surfaces form the Smad-Smad binding interface in the Smad trimer. Although the Smad MH2 domain is able to bind many different Smad-interacting proteins, these interacting proteins do not share a conserved sequence motif that binds to Smad MH2. Structural analyses of R-Smad co-crystals have defined binding sites for just three R-Smad protein-protein interactions: the Smad-Smad trimer interface [20], [21], [22], [23], Smad4 binding to a peptide from Ski [31], and the hydrophobic groove on Smad2 that binds to a peptide from SARA [22], [32], [33].

We have generated a series of Smad3 variants that are selectively compromised in binding a subset of Smad3-interacting proteins by engineering mutations in one or more amino acids on the surface of Smad3 at putative protein-binding “hot-spots” [34], [35], [36], [37]. Hot-spots may be clustered on the protein surface, producing hot regions; the hot-spots within a hot region may exhibit cooperative binding to a protein ligand [38], [39]. Different hot regions are used to bind to different partners in a hub protein [4]. Amino acids were selected for mutation based on their apparent role in mediating protein binding in one of the known Smad co-crystal structures, by their location on the surface of the Smad3 MH2 domain crystal structure and enrichment in hot-spots in other proteins [35], [40], [41], [42], [43], or because of previous mutagenesis studies that showed reductions in Smad3 or Smad2 protein binding. For example, mutations in the alpha-helix2 region within the hydrophobic groove were previously shown to reduce the binding interaction with the Smad-binding domains from the Xenopus FoxH1 and Mixer transcription factors [44], [45]. Results are presented for 28 variants distributed across three different regions on the surface of Smad3: the interfaces mediating binding to Smad4, the hydrophobic corridor defined by the Smad2-SARA co-crystal structure, and the cap region defined by its role in binding to Ski. We characterized the Smad3 variants for binding to a panel of candidate Smad3-binding proteins and peptides all of which had been shown to bind directly to Smad3 in previous studies. Most of the Smad3 variants exhibited reduced binding to one or more of the seventeen Smad3-binding proteins or peptides tested, consistent with the mutated amino acids contributing significantly to one or more binding interactions, i.e, a hot-spot, but also consistent with the presence of different amino acids on Smad3 acting as hot-spots for binding to different protein or peptide ligands. The functional consequences of the mutations were assayed by quantifying their ability to reconstitute TGF-β-induced, Smad3-dependent reporter gene activation in Smad3-deficient JEG-3 cells [46] and by their affects on the TGF-β-inducibilty of six Smad3-regulated genes in C2C12 myoblasts. E267A increased protein binding and had the unique property of reducing TGF-β inducibility by inducing high basal expression levels of the six endogenous genes. Y297A fully rescued reporter gene expression but mediated reduced expression of the six endogenous genes while unexpectedly increasing the fold activation of these genes by TGF-β because it induced lower basal expression levels of the genes than did wild-type Smad3. Y226A reduced reporter gene expression by only 50% but was comparable to W406A and V356R in its severe reduction of the TGF-β-inducibilty of the six endogenous genes. As with Y226A, K341A reduced reporter gene activation by about 50% but in contrast to Y226A it only had minimal effects on the six endogenous genes except for reducing expression levels of Olfm2. The double mutant Q322A/Y324A was as severe as W406A in reducing the ability of Smad3 to increase the expression of the six endogenous genes but retained more function than W406A in mediating the reporter gene activation and in the TGF-β-inducibilty of the six Smad3-regulated genes in C2C12 myoblasts.

## Results

**Figure 1:**
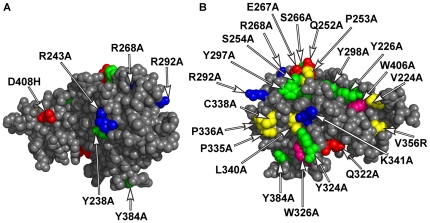
Mutations on the Smad3 MH2 domain. (A) A model of the Smad3 MH2 domain showing the surfaces including the triple helix and loop helix interfaces that mediate binding to Smad4 [23] with mutated amino acids highlighted. (B) A model of the Smad3 MH2 domain rotated 180° from the view in panel A, showing the surface with the hydrophobic corridor that binds to SARA [32] with mutated amino acids highlighted: tryptophan in hot-pink; aspartic acid, glutamic acid and glutamine in red; arginine and lysine in blue; tyrosine and serine in green; and proline, valine, and leucine in yellow.

Amino acids predicted by the crystal structure to be on the surface of the Smad3 MH2 domain were mutated, typically to alanine (Figure 1). These amino acids were chosen based on prior studies implicating them in mediating Smad3 or Smad2 protein interactions, or because they are among the group of amino acids (R, W, Y) enriched in mediating protein-protein interaction hot-spots in other proteins [35]. Prior structural and mutational studies on Smad2 were used to guide our mutational analysis of Smad3 because Smad3 and Smad2 MH2 domains share 92% identity at the primary amino acid sequence level and similar 3D structures [23]. Mutations were made on the triple helix and loop-helix interfaces, respectively, that mediate Smad3 binding to Smad4 or Smad2 in the heterotrimeric structure formed upon phosphorylation of Smad3 (Figure 1A) [20], [21], [22], [23]. Most of the mutations were made on the opposite side of Smad3 that includes the hydrophobic corridor defined by the co-crystal of the SARA Smad-binding domain and Smad2 [32](Figure 1B). Mutations were also made in a region at the top of the Smad trimer toroid previously shown to be involved in binding to Ski [47](Figure 1B).

The effect of any one mutation or pair of mutations on Smad3 protein binding was quantified using a modification of the LUMIER (luminescence-based mammalian interactome mapping) assay originally described by Dr. Jeffery Wrana and colleagues [48]. The full-length mutant Smad3 proteins were expressed as Renilla luciferase fusion proteins in HEK-293 cells together with a Flag-tagged Smad3 binding partner and the constitutively activated Alk5 Type I receptor kinase that phosphorylates Smad3. The Flag-tagged Smad-binding partners were expressed either as full-length proteins or as Smad-binding domains displayed on the surface of a Thioredoxin (Trx) scaffold. We have previously described the use of the Thioredoxin scaffold to display Smad-binding domains [49], [50], [51]. The Flag-tagged protein was recovered from cell lysates on anti-Flag antibody coated 96-well plates and the amount of co-immunoprecipitated Smad3 was measured by Renilla luciferase activity. Co-expression of the activated Alk5 kinase led to increased binding of Renilla-Smad3 to Flag-Smad4 (Table 1), confirming that the binding assay accurately detected the increased binding of Smad3 to Smad4 expected upon phosphorylation of Smad3. In contrast, Smad3 binding to the Smad-binding domain from SARA was not significantly affected by co-transfection with the activated receptor kinase (Table 1), consistent with the ability of the SARA Smad-binding domain to bind unphosphorylated or phosphorylated Smad3 [51].

**Table 1:** The luminescence-based mammalian interactome assay (LUMIER) detects reductions in subsets of Smad3 protein interactions due to mutations in Smad3 or the absence of Smad3 phosphorylation.

Smad3 (WT or mutant)	Percent binding relative to the binding by phosphorylated wild-type Smad3			
	Smad4	SARA	Smurf2	Ski
	FL	aa665–721	aa252–330	aa2–56
**Smad3 WT+Alk5**	**100**	**100**	**100**	**100**
**Smad3 WT, No Alk5**	**6** (1)	**78** (26)	**Nd**	**nd**
**D408H**	**3** (1)	**125** (45)	**23** (12)	**3** (1)
**Del-PPGY (182–185)**	**120** (15)	**129** (16)	**3** (1)	**160** (50)
**Y297A**	**181** (42)	**8** (3)	**119** (20)	**1** (1)
**QPSMT/SE** *	**132** (18)	**61** (26)	**86** (29)	**4** (1)

Binding between wild-type or mutant Renilla-Smad3 fusion proteins and four different Flag epitope-tagged Smad-binding proteins was quantified by pull-down of protein complexes from cell lysates in anti-Flag antibody-coated 96-well plates and measuring the amount of luciferase activity per well in a luminometer. The luciferase counts were normalized to the number of counts recovered with phosphorylated wild-type Smad3 (100%). All of the Smad3 mutant proteins were co-expressed with the constitutively active Alk5. The average of the data from at least four independent transfections is shown. Total Renilla luciferase activity was measured in each transfected cell lysate as a surrogate measure for the level of Renilla-Smad fusion protein; data is only reported for experiments where the level of each Renilla-Smad3 mutant was within two-fold of the level of the Renilla-Smad3 wild type protein in the same experiment. FL indicates that the full-length Smad4 protein was used. The amino acids comprising the SARA [32], Smurf2 [52] and Ski [22] Smad3-binding motifs that were expressed on the Flag-epitope tagged thioredoxin scaffold are indicated below each gene. The reduced binding interactions of the Smad3 mutants D408H and Y297A to the Ski Smad-binding domain are consistent with previous studies using different assay methods [22]. Standard deviations are indicated in parenthesis. Not determined (nd).

*The Smad3 QPSMT/SE mutant has residues 252–256 and 266–267 changed to the Smad1 equivalent amino acids (STSVL/NKN) [47].

### Mutations known to disrupt Smad3 binding interactions are detected in the LUMIER assay and exhibit selective disruption of a subset of binding interactions

Retention of control levels of binding to one or more Smad-binding proteins by the mutant Smad3 proteins was used as the criteria to indicate that a specific mutation was exerting a local effect and not causing a general disruption of Smad3 MH2 protein structure/folding. Mutations in amino acids that reduced co-immunoprecipitation with all of the Smad-binding proteins tested (F303A, R295A and R240A) were likely to have affected the overall structure of the MH2 domain and were not included in further analyses. Reduced binding by a Smad3 mutant to any single Smad-binding protein, relative to the binding of wild-type Smad3, also could be an artifact of low expression of the mutant protein. To control for this, total Renilla luciferase activity was measured in each transfected cell lysate as a surrogate measure for the level of Renilla-Smad fusion protein. Co-immunoprecipitation data is only reported for experiments where the level of luciferase activity of the mutant Renilla-Smad3 fusion protein was within two-fold of the level of the luciferase activity of the wild-type Renilla-Smad3 fusion protein in the same experiment.

Four mutant Smad3 proteins each previously reported to be defective in binding to one Smad-binding partner were co-expressed with Smad4 or one of three different Smad-binding peptides to determine if the assay was sensitive enough to detect perturbation of binding by mutations in Smad3 (Table 1). The three Smad-binding peptides were from SARA [32], Smurf2 [52] and Ski [22], [53]. Mutation of Smad3 D408 to histidine (D408H) greatly reduced binding to Smad4, consistent with previous studies in which mutation of D408 in Smad3, or the corresponding aspartic acid in Smad2 or Smad4, resulted in defects in Smad complex formation and in TGF-β-dependent phosphorylation of the mutant R-Smad [54], [55], [56], [57]. Smad3 D408H retained wild-type levels of binding to the SARA Smad-binding peptide, however, binding to the Smad-binding peptides from Smurf2 and Ski was significantly reduced (Table 1); the reduction in binding to Ski was consistent with prior studies on this mutation [22]. Deletion of the PPGY motif in the Smad3 linker region that mediates the interaction between R-Smads and the Smurf2 WW domains [52], [58] reduced binding specifically to the Smad binding domain from Smurf2 (Table 1). Mutation of Y297 to alanine reduced binding to the SARA Smad-binding domain, as predicted by the co-crystal structure [32], and to the Smad-binding domain from Ski [22] but it did not reduce binding to Smad4 or to the Smad-binding peptide from Smurf2 (Table 1). Smad3 with the STSVL of Smad1 in place of QPSMT of Smad3 amino acids 252–256 and the NKN residues of Smad1 in place of the SE of Smad3 amino acids 266–267 exhibited decreased binding to the Smad-binding peptide from Ski as reported in previous studies [47], but retained binding to Smad4 and the Smurf2 Smad-binding peptide (Table 1). Since the assay was able to detect selective perturbations on protein binding imparted by the control mutations in Table 1, it was used to examine a larger number of mutations and interacting proteins.

### Smad3 mutations that reduce binding to Smad4 also reduce other protein-protein interactions

The six mutations in Smad3 shown in Figure 1A reduced binding to Smad4 (Table 2). With the exception of R292, these have all been implicated by the co-crystal structures in mediating the Smad3-Smad4 interaction [21], [23]. D408 and R268 form salt bridges between Smad3 and the corresponding arginine or aspartic acid, respectively, on Smad4 or an R-Smad in the heterotrimer. Smad3 R243 participates in a charge-charge interaction with E337 on Smad4 and the position of Y238 in the structure indicates that it could be involved in the proper positioning of R243. All six mutations also reduced binding to Smad3, but retained binding to the SARA Smad-binding domain and full length SARA [59]. The Smad3 D408H mutation also retained near wild-type levels of binding to the xFoxH1, SRF and ATF3 Smad-binding peptides, MyoD, MEF2C, and Axin (Table S1). All six mutations unexpectedly reduced binding to the Smad-binding domains from Smurf2 and SIP1 (Table 2). Binding to the Ski Smad-binding peptide was greatly reduced by the six mutations, but this was consistent with previous reports in which binding of the Ski peptide was reduced in R-Smad mutants unable to form hetero-trimeric complexes [22]. The W274E mutation of full-length Ski, which reduces binding of Ski to Smad4 [53] was used to limit the binding interaction detected in the co-immunoprecipitation assay to Smad3-Ski. Binding of the SkiW274E protein to Smad3 also was reduced by the six mutations but there was significantly more binding to full-length Ski than to the Ski Smad-binding peptide. The binding behavior of all six mutants was highly correlated; the Spearman's rank correlation coefficients were from 0.66 to 0.097 and all pairwise correlations were significant (p<0.05).

**Table 2:** Mutations on the Smad3 MH2 interfaces that mediate binding to Smad4 reduce binding to Smad4 or Smad3 but have differential effects on interactions with other Smad-binding proteins.

Smad3 Mutants	Percent binding to Smad3 mutants relative to the binding to wild-type Smad3 (100%)							
	Smad4	Smad3	SARA	SARA	Smurf2	Ski	SkiW274E	Sip1
	FL	FL	665–721	FL	252–330	2–56	FL	315–487
**D408H**	**3** (1)	**30** (5)	**125** (45)	**168** (52)	**23** (12)	**3** (1)	**31** (7)	**41** (9)
**R268A**	**18** (8)	**27** (8)	**116** (14)	**159** (40)	**21** (9)	**2** (2)	**62** (28)	**13** (10)
**Y238A**	**27** (9)	**24** (2)	**74** (4)	**162** (50)	**28** (9)	**2** (1)	**44** (3)	**15** (2)
**R243A**	**20** (14)	**46** (1)	**72** (33)	**181** (24)	**19** (3)	**3** (1)	**72** (16)	**28** (3)
**R292A**	**49** (16)	**49** (16)	**78** (31)	**120** (33)	**31** (16)	**4** (1)	**59** (11)	**32** (2)
**Y384A**	**19** (9)	**28** (6)	**119** (44)	**178** (33)	**24** (11)	**3** (1)	**53** (6)	**41** (6)

Binding between wild-type or mutant Renilla-Smad3 fusion proteins and eight different Flag epitope-tagged Smad-binding proteins was quantified by pull-down of protein complexes from cell lysates and detection of the Renilla luciferase activity per well. The Renilla luciferase counts were normalized to the number of counts recovered with phosphorylated wild-type Smad3 (100%). The proteins were co-expressed with the constitutively active Alk5. The average of the data from at least four independent transfections is shown. Total Renilla luciferase activity was measured in each transfected cell lysate as a surrogate measure for the level of Renilla-Smad fusion protein; data is only reported for experiments where the level of each Renilla-Smad3 mutant was within two-fold of the level of the Renilla-Smad3 wild type protein in the same experiment. FL indicates that the full-length protein was used, e.g., full-length SARA [59]. The amino acids comprising the SARA [32], Smurf2 [52], Ski [22], and Sip1 [94] Smad3-binding motifs expressed on the Flag-epitope tagged thioredoxin scaffold are indicated below each gene. Standard deviations are indicated in parenthesis.

### Smad3 mutations that retain binding to Smad4 are defective in binding to other partners

The co-crystal structure of Smad2 and the SARA Smad-binding domain defined a hydrophobic corridor on the surface of Smad opposite the interfaces mediating binding to Smad4 [32] (Figure 1B). The Smad3 mutations in Table 3 fall in or near this hydrophobic corridor. These fourteen Smad3 mutations retained binding to Smad4 and Smad3 but exhibited altered binding to other proteins tested (Table 3). All of the mutations had wild-type or greater binding to the Smad-binding domain from Smurf2, in contrast to the deletion of the PPGY motif that reduced binding to the Smurf2 Smad-binding peptide (Table 3). The mutations in the region of the hydrophobic corridor near the Smad3 alpha helix 2, which is on the lower left side in Figure 1B, Q322A/Y324A and W326A decreased binding to SARA (Table 3). These amino acids were previously reported to be involved in binding to SARA [32] and are also involved in binding a motif from the *Xenopus* proteins FoxH1, Mixer and Milk [44] (Table S1). However, these mutations did not reduce binding to the Smad-binding domains from Ski or SIP1 (Table 3).

**Table 3:** Mutations on the Smad3 MH2 interface that mediates binding to SARA [32] retain binding to Smad4 and Smad3 but have differential effects on interactions with other Smad-binding proteins including, in some cases, increased binding.

Smad3 Mutants	Percent binding to Smad3 mutants relative to the binding to wild-type Smad3 (100%)							
	Smad4	Smad3	SARA	SARA	Smurf2	Ski	SkiW274E	Sip1
	FL	FL	665–721	FL	252–330	2–56	FL	315–487
**Q322A/Y324A**	**124** (6)	**143** (12)	**3** (1)	**12** (5)	**308** (44)	**237** (42)	**100** (22)	**162** (29)
**W326A**	**194** (20)	**159** (46)	**4** (3)	**13** (6)	**653** (198)	**292** (73)	**88** (15)	**319** (38)
**P335A/P336A**	**309** (59)	**109** (9)	**2** (1)	**16** (5)	**255** (79)	**356** (55)	**118** (28)	**123** (24)
**C338A**	**125** (38)	**100** (17)	**94** (11)	**136** (23)	**153** (21)	**188** (32)	**111** (42)	**44** (11)
**L340A**	**181** (42)	**99** (31)	**12** (3)	**58** (10)	**259** (51)	**194** (60)	**57** (19)	**159** (16)
**K341A**	**275** (60)	**95** (18)	**4** (2)	**29** (4)	**130** (30)	**27** (8)	**55** (15)	**66** (8)
**S254A**	**101** (46)	**105** (10)	**103** (32)	**154** (34)	**113** (28)	**10** (5)	**78** (25)	**128** (103)
**E267A**	**458** (399)	**222** (12)	**81** (8)	**82** (20)	**379** (71)	**1152** (374)	**137**(9)	**88** (41)
**Y297A**	**181** (42)	**82** (10)	**8** (3)	**38** (11)	**119** (20)	**1** (1)	**29** (10)	**80** (7)
**Y298A**	**120** (13)	**113** (21)	**73** (26)	**150** (47)	**144** (7)	**271** (73)	**121** (19)	**33** (19)
**V224A**	**122** (13)	**118** (26)	**21** (9)	**42** (5)	**320** (59)	**524** (84)	**212** (63)	**339** (114)
**Y226A**	**110** (9)	**161** (26)	**3** (1)	**32** (10)	**365** (26)	**32** (17)	**89** (14)	**7** (6)
**V356R**	**233** (55)	**275** (87)	**1** (1)	**16** (12)	**468** (70)	**2** (8)	**30** (4)	**7** (7)
**W406A**	**109** (8)	**564** (89)	**1** (1)	**12** (3)	**1327** (151)	**5** (1)	**21** (7)	**15** (1)
**Del-PPGY**	**120** (15)	**86** (15)	**129** (16)	**158** (69)	**3** (1)	**160** (50)	**83** (18)	**200** (13)

Binding between wild-type or mutant Renilla-Smad3 fusion proteins and eight different Flag epitope-tagged Smad-binding proteins was quantified by pull-down of protein complexes from cell lysates and detection of the Renilla luciferase activity per well. The Renilla luciferase counts were normalized to the number of counts recovered with phosphorylated wild-type Smad3 (100%). The proteins were co-expressed with the constitutively active Alk5. The average of the data from at least four independent transfections is shown. Total Renilla luciferase activity was measured in each transfected cell lysate as a surrogate measure for the level of Renilla-Smad fusion protein; data is only reported for experiments where the level of each Renilla-Smad3 mutant was within two-fold of the level of the Renilla-Smad3 wild type protein in the same experiment. FL indicates that the full-length protein was used. The amino acids comprising the SARA [32], Smurf2 [52], Ski [22], and Sip1 [94] Smad3-binding motifs expressed on the Flag-epitope tagged thioredoxin scaffold are indicated below each gene. The Smad3 Del-PPGY (182–185) mutation is included in the last row although it resides in the Smad3 linker region and not in the MH2 domain. Reduced binding interactions to the Smad interaction motif from Ski by W406A, Y297A and Y226A are consistent with previous studies on this protein interaction that used size exclusion chromatography with purified proteins [22], [64]. Standard deviations are indicated in parenthesis.

Four mutations were created in the beta-8/beta-9 region of Smad3, which is on the left side in Figure 1B: P335A/P336A, C338A, L340A, and K341A [63] (Figure 1B and Table 3). The P335A/P336A double mutant was selective for only decreasing binding of the SARA Smad-binding domain and full-length SARA of the proteins tested. Given that Smad3 binds to over 50 proteins, it seems likely that this double mutant will be defective in binding to other proteins in addition to SARA, but we do not know the identity of these at this time. Similarly, Smad3 C338A was selective for a partial but significant reduction in binding to the SIP1 Smad-binding domain. The L340A mutation reduced binding to SARA and to SkiW274E, but not to the Smad-binding aa2-56 peptide from Ski. The K341A mutation had broader effects on protein binding, reducing binding to the Smad-binding domains from SARA, Ski and SIP1 as well as the full-length proteins SARA and SkiW274E. The Smad3 K341A mutation maintained substantial binding interactions with several other proteins tested (Table S1). Double mutations of the corresponding cysteine and leucine or leucine and lysine in Smad2 were reported to interfere with Smad2 nucleocytoplasmic shuttling [63].

A Smad1/3 chimeric protein expressing the NKN/STSVL residues of Smad1 in place of the SE/QPSMT amino acids in Smad3, exhibited decreased binding to Ski [47]. Each of the six amino acids in this region, which is on the top of the structure shown in Figure 1B, that are different between Smad1 and Smad3 were mutated to alanine in Smad3. None of the mutations had decreased binding to Smad3 or Smad4 (Table 3 and Table S2). Of these six mutations, only Smad3 S254A decreased Smad3 binding to the Ski Smad-binding domain (Table 3). Unexpectedly, E267A had increased binding to Smad3, Smad4, and the Smad-binding domains from Ski and Smurf2 (Table 3). An adjacent mutation, Y297A [22] (Figure 1B), caused reduced binding to the Ski Smad-binding domain and SkiW274E, but also caused significant reductions in binding to the SARA Smad-binding domain and SARA (Table 3).

Five mutations were made in the region of the hydrophobic corridor formed in part by the triple helix bundle, located on the right side of Figure 1B: Y298A, V224A [22], [64], Y226A [22], [64], V356R [63], [65] and W406A [22], [63], [64], [65]. The Y298A mutation caused a significant reduction specifically in binding to the SIP1 Smad-binding domain whereas the V224A mutation selectively reduced binding to the SARA Smad-binding domain and SARA. The Y226A, V356R and W406A mutations reduced binding to full length SARA as well as the SARA, Ski and SIP1 Smad binding domains (Table 3). The Smad3 mutations V356R and W406A (and the corresponding mutations in Smad2) also are known to interfere with Smad2 nucleocytoplasmic shuttling [63], [65]. V224A, Y226A, V356R and W406A Smad3 variants exhibited near wild-type levels of binding to MyoD, MEF2C, Axin and the SRF Smad-binding domain (Table S1).

### Transcriptional activation of a TGF-β-responsive, Smad3-dependent reporter gene is compromised by some Smad3 mutations

**Figure 2:**
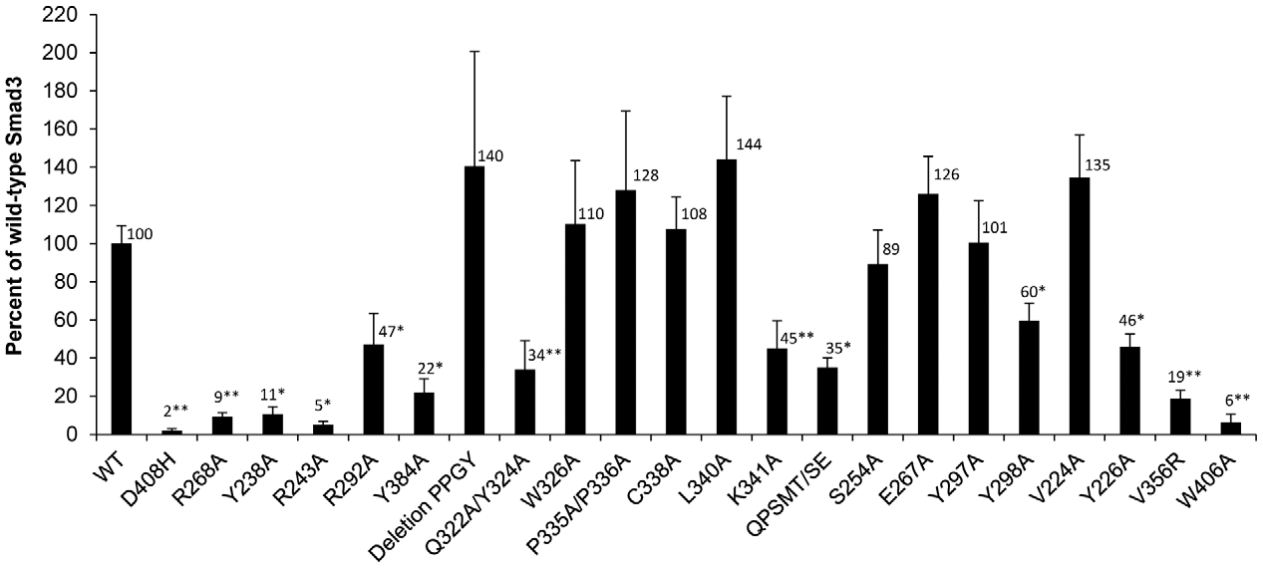
Rescue of TGF-β signaling in Smad3 deficient JEG-3 cells by wild-type and mutant Renilla-Smad3 fusion proteins. JEG-3 cells were transfected with plasmids for the Smad3-dependent firefly luciferase reporter gene SBE12-luciferase [97], the pCMV5 Renilla luciferase plasmid encoding wild-type or mutant Smad3 and a plasmid encoding beta-galactosidase that was used to normalize for differences in transfection efficiency. After 24 hours of 100 pM TGF-β, cell lysates were analyzed for luciferase and beta-galactosidase activities. Total Renilla luciferase activity was measured in each transfected cell lysate to confirm comparable expression levels of Renilla-Smad3 fusion proteins. The TGF-β-induced firefly luciferase activity for each mutant was normalized to the activity provided by wild-type Smad3 (100%). The average of the data from a minimum of six independent transfections with each Smad3 variant are shown. The levels of reporter gene expression for cells transfected with wild-type and mutant Smad3 were compared using a permutation test that combined the results of the replicate experiments. For mutants with reduced reporter gene activity, P-values (corrected for multiple comparisons) less than 0.02 (*) and less than 0.001 (**) are indicated. The activity observed with Smad3 D408H was indistinguishable from the activity of JEG-3 cells without any exogenous Smad3. Most of the Smad3 mutants that altered binding to Smad4 (Table 2) provided reduced rescue of Smad3 signaling. Some of the mutations that retained Smad4 binding also provided reduced rescue of Smad3 signaling but other Smad3 mutations that had specific defects in protein binding (Table 3) were as effective as wild-type Smad3 for restoring signaling in the JEG-3 cells.

The Smad3 variants were expressed in the Smad3-deficient JEG-3 choriocarcinoma cell line [46] together with a Smad3-dependent luciferase reporter plasmid. Expression of wild-type Smad3 and several of the mutant Smad3 proteins restored TGF-β activation of the reporter gene (Figure 2). The D408H mutant protein and the other mutants shown in Table 1 that had reduced Smad4 binding, all failed to restore signaling, consistent with the canonical Smad pathway in which Smad3 (and Smad2) form heterotrimers with Smad4 to activate or repress target gene expression. Of the six mutations in Table 2, the R292A mutant had the greatest binding to Smad4 and the highest rescue of reporter gene expression (Figure 2). As the mutations are listed in Figure 2 by their location on the structure (Figure 1), note the uniform structure-activity response of mutations on the Smad4-binding interface (D408 to Y384) versus the dispersed effect of mutations elsewhere on the Smad3 surface. For the Smad3 variants that bound to Smad4 (Table 3), some had wild-type levels of transcriptional activity on the reporter gene, while several others, Q322A/Y324A, K341A, Y298A, Y226A, V356R and W406A, were compromised in their ability to activate transcription from the reporter gene. In terms of structure-activity relationships, Y324 and K341 may define a motif that appears to functionally important, although several other amino acids in this region, e.g., L340 and W326, were not required for reporter gene activiation. A second functional region may be defined by Y226, V356 and W406, although V224, which resides right in the middle of the other three, was not required. A previous report showed that the Smad3 mutants V356R and W406A failed to restore TGF-β-induced reported gene activity in HaCaT human kerationcytes, consistent with the defects these mutations cause in Smad3 nucleocytoplasmic shuttling [65]. The other mutations may reduce the binding of Smad3 to one or more Smad-binding proteins such that the reporter gene is not fully induced by TGF-β in the JEG-3 cells, however these studies do not identify which Smad3-binding protein or proteins are needed for full reporter gene activation in the JEG-3 cells.

The ability of each Smad3 mutant to rescue the reporter gene was correlated with the protein binding data in Tables 2 and 3 using Kendall's rank correlation analysis (Table 4). As expected, the analyses revealed significant correlation of the Smad3 mutants binding to Smad3 and Smad4 and correlation of the Smad3 mutants binding to either the peptide binding motif or full length proteins of SARA or SKI. Unexpectedly, the binding of the panel of hot-spot mutations correlated significantly for binding to both peptide motifs from the Smad3 transciptional co-factor SIP1 and the Smad3 inhibitor SKI (Table 4). Significant negative correlations indicated that hot-spots reducing binding to one of these Smad3 binding partners correlated with increased binding to another Smad3 binding partner. As expected, rescue of the Smad3-dependent reporter gene activation correlated with binding to Smad4, consistent with the canonical Smad3 signaling pathway in which a Smad3-Smad4 heterotrimer activates transcription. Rescue also significantly correlated with how the entire panel of hot-spot mutations affected binding to the SIP1 peptide or SKI274E. It is important to note that these results do not imply that SIP1 or SKI are involved in activating the Smad3-dependent reporter gene in JEG3 cells. SKI, for example, is a negative regulator of Smad3 signaling, not a positive co-factor. Rather, the results imply that the constellation of hot spots on Smad3 involved in optimal binding to the SIP1 peptide and to SKI are also involved in binding to un-identified co-factors in the JEG3 cells that are needed for optimal Smad3-dependent activation of the luciferase reporter gene. In contrast, rescue of the reporter gene did not correlate with how the whole panel of hot-spot mutations affected binding to Smurf2 peptide or to SARA, even though there were individual mutations, e.g., Smad3 W406A, that reduced both binding to SARA (Table 3) and rescue of the reporter gene (Figure 2). The different conclusions reached by correlation analysis of the entire panel of hot-spot mutations versus the properties of a single mutation, Smad3W406A, indicates that correlation analysis of the protein binding and functional activity of a large panel of hot-spot mutations may be a powerful approach to identify which endogenous Smad3 binding proteins are involved in specific gene expression responses.

**Table 4:** Correlation analysis of Smad3 mutant protein binding assays and rescue of the Smad3-dependent reporter gene assay.

Sample1	Sample2	tau	P-value	FDR
Smad3	Smad4	0.4	0.005045	0.01261
TrxSki	SkiW274E	0.64	0.0001	0.0002
SARA	TrxSARA	0.54	0.0001	0.0016
TrxSki	TrxSIP1	0.48	0.0008	0.0036
SARA	TrxSmurf2	−0.46	0.0012	0.0048
Smad4	TrxSARA	−0.32	0.0247	0.0464
RESCUE	Smad4	0.46	0.0013	0.0044
RESCUE	TrxSIP1	0.56	0.0001	0.0014
RESCUE	SkiW274E	0.4233	0.003008	0.008461
RESCUE	SARA	−0.003333	0.9814	0.9814
RESCUE	TrxSmurf2	0.2267	0.1123	0.1742

Sample1	Sample2	tau	P-value	FDR
Smad3	Smad4	0.4	0.005	0.013
Ski 2–56	SkiW274E	0.64	0.0001	0.0002
SARA	SARA 665–721	0.54	0.0001	0.0016
Ski 2–56	SIP1 315–487	0.48	0.0008	0.0036
SARA	Smurf2 252–330	−0.46	0.0012	0.0048
Smad4	SARA 665–721	−0.32	0.025	0.046
RESCUE	Smad4	0.46	0.0013	0.0044
RESCUE	SIP1 315–487	0.56	0.0001	0.0014
RESCUE	SkiW274E	0.42	0.003	0.0085
RESCUE	SARA	−0.003	0.98	0.98
RESCUE	Smurf2 252–330	0.23	0.11	0.17

Protein binding data from Tables 2 and 3 and reporter gene activation data (RESCUE) from Table 2 were tested pairwise for their ability to correlate based on each sample's results with all of the Smad3 mutants using Kendall's rank correlation analysis. The data were analyzed using Mstat 5.4. Tau is the correlation coefficient. The p-value is shown with the false discovery rate (FDR) correction, which accounts for the multiple comparisons. The first three rows of data confirm expected results that, for the panel of hot-spot mutations examined, binding to Smad3 and Smad4 correlate, binding to full length SkiW274E and the Ski peptide correlate, and binding to full length SARA and the SARA peptide correlate. There is a positive correlation, not predicted by any prior studies, that a similar pattern of Smad3 hot-spots are involved in binding to both the SKI peptide and the SIP1 peptide. In contrast, there are significant negative correlations between Smad3 mutants binding to SARA versus Smurf2 peptide as well as between Smad3 mutants binding to Smad4 versus SARA peptide. The analysis also was used to correlate the protein binding properties of the hot spot mutations (LUMIER assay in 293 cells, Tables 2 and 3) to function (reporter gene assay in JEG3 cells, Figure 2). As expected, rescue of the Smad3-dependent reporter gene activation correlated with binding to Smad4. Rescue also significantly correlated with how the hot-spot mutations affected binding to the SIP1 peptide or SKI274E. In contrast, rescue of the reporter gene did not correlate with how the Smad3 mutant hot-spots affected binding to Smurf2 peptide or to SARA.

### Smad3 variants exhibit quantitative differences in the TGF-β inducibilty of six Smad3-regulated genes in C2C12 cells

**Figure 3:**
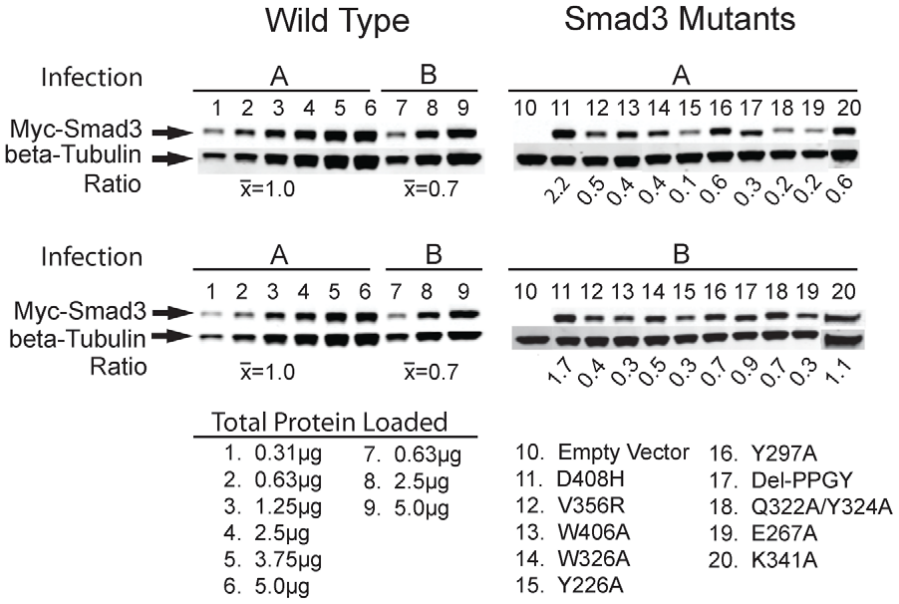
Comparable expression levels of wild-type myc-Smad3 and myc-Smad3 variants in infected C2C12 cell populations. Cell lysates were prepared from two independent sets (A and B) of C2C12 cell populations independently infected with retrovirus encoding wild-type myc-Smad3 (blots on the left) and either mutant myc-Smad3 genes or retrovirus without the myc-Smad3 gene (Empty Vector, EV, blots on the right). The lysate proteins were resolved by SDS-PAGE. Western blots were probed with anti-myc antibody and IRDye goat anti-mouse 800 secondary antibody, as well as anti-beta-Tubulin antibody and IRDye goat anti-rabbit 680 secondary antibody and imaged on an Odyssey IR scanner. Titrations of wild-type Smad3 lysates from both infections were analyzed two times (left-top and left-bottom of figure) and were used to generate a standard curve. Total lysate protein (2.5 µg) was loaded for lysates from cells infected with EV or mutant Smad3 retroviruses with the exception of K341A (1.25 µg, lane 20, infection B). The lack of signal in the EV cell lysates loaded in lane 10 of each gel indicates that the signal being detected is myc-Smad3. The ratio represents the expression of myc-Smad3 relative to the beta-Tubulin loading control. The “Infection A” set had expression of mutant Smad3's within a ten-fold range of wild-type myc-Smad3; the “infection B” set had expression of mutant Smad3's within a three-fold range of wild-type myc-Smad3. The Smad3 D408H mutation (lane 11) consistently had the highest levels of Smad3 protein expression.

To examine whether the individual Smad3 mutations modify the ability of the protein to mediate TGF-β induction of endogenous genes, myc-epitope-tagged Smad3 variants or wild-type protein were over-expressed from a retroviral vector in populations of infected C2C12 myoblasts. Levels of wild-type and mutant Smad3 proteins were quantified in two independent replicates (A and B) of infected C2C12 cell populations by quantitative Western blots using fluorescent secondary antibodies and standard curves as described in the Materials and Methods (Figure 3). The expression levels of myc-Smad3 variants relative to beta-tubulin expression levels were generally lower than the wild-type myc-Smad3 expression in the C2C12 populations although at least one replicate cell population for each variant was within three-fold of the wild-type Smad3/beta-tubulin ratios of 1.0 (A) and 0.7 (B).

**Figure 4:**
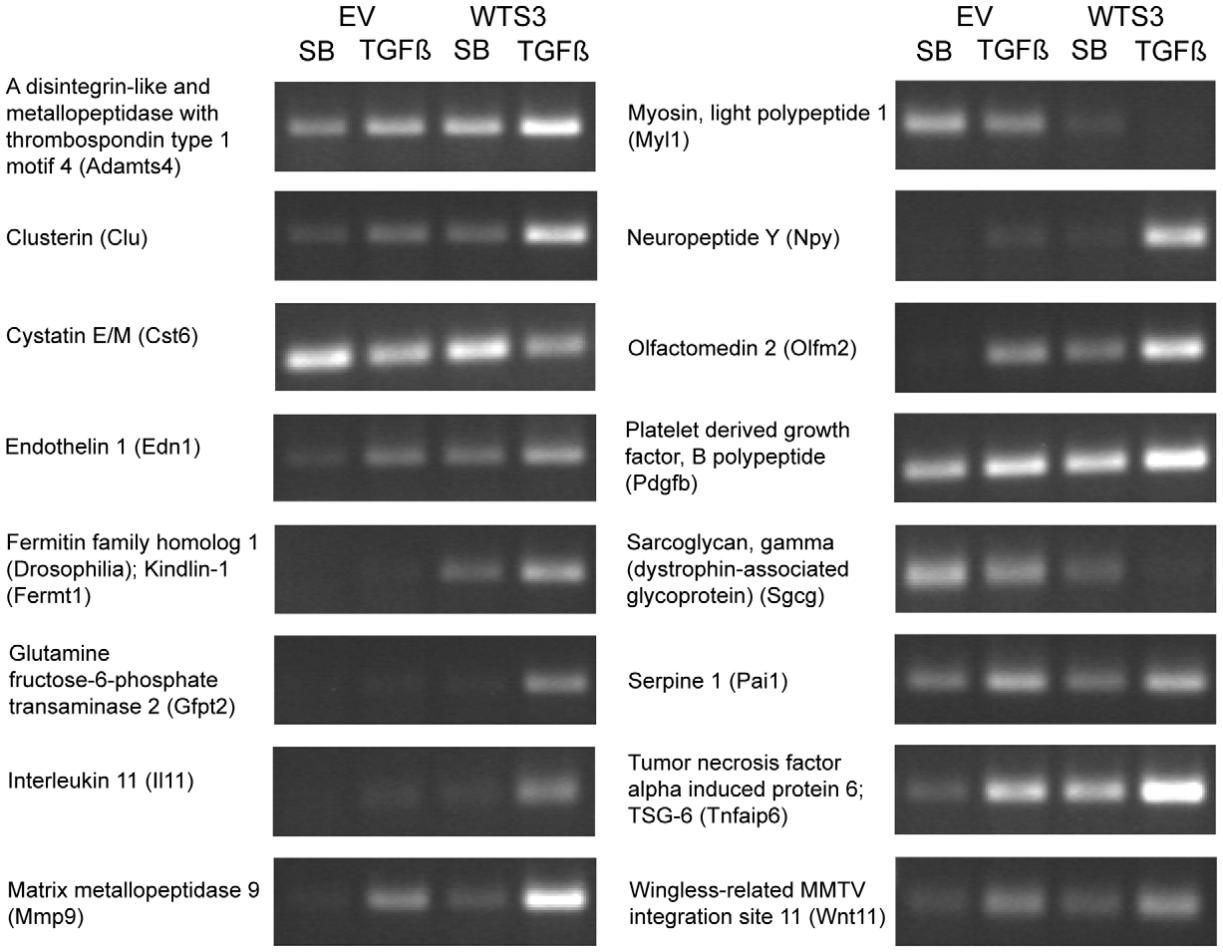
Smad3 and TGF-β alter the levels of gene expression in C2C12 cells. C2C12 cell populations infected with retrovirus without the myc-Smad3 gene (Empty Vector, EV) or retrovirus encoding wild-type Smad3 were treated with either 1 µM SB431542 or 100 pM TGF-β for 24 hours before RNA isolation. The 16 genes were amplified by RT-PCR using the primer sets shown in Table S3. The annealing temperature and number of cycles of PCR were optimized for each primer set. The overexpression of Smad3 had modest effects on the levels of some genes (Adamts4, Cst6, Pdgfb, Pai1 and Wnt11) but had detectable effects on the levels of other genes (Fermt1, Gfpt2, Il11, Mmp9, Myl1, Npy, Olfm2, Sgcg). The expression levels of three genes, Cst6, Myl1 and Sgcg, were decreased by TGF-βtreatment. Overexpression of Smad3 caused further reductions in the levels of Myl1 and Sgcg.

Several candidate Smad3-induced genes were identified from the literature or identified on gene arrays with mRNA from TGF-β-treated C2C12 cells infected with the retrovirus expressing wild-type Smad3 (see Materials and Methods); changes in gene expression were confirmed using RT-PCR (Figure 4). Several genes showed increased basal expression with addition of the exogenous Smad3 from the retroviral infection and an additional increase upon treatment with TGF-β for 24 hours. Note, for example, the brighter band intensity for Adamts4 with addition of exogenous Smad3 and the further increase in brightness with TGF-β treatment (Figure 4). We also identified three genes whose expression was decreased by the addition of exogenous Smad3 and TGF-β treatment in C2C12 cells: Cst6, Myl1 and Sgcg (Figure 4).

**Figure 5:**
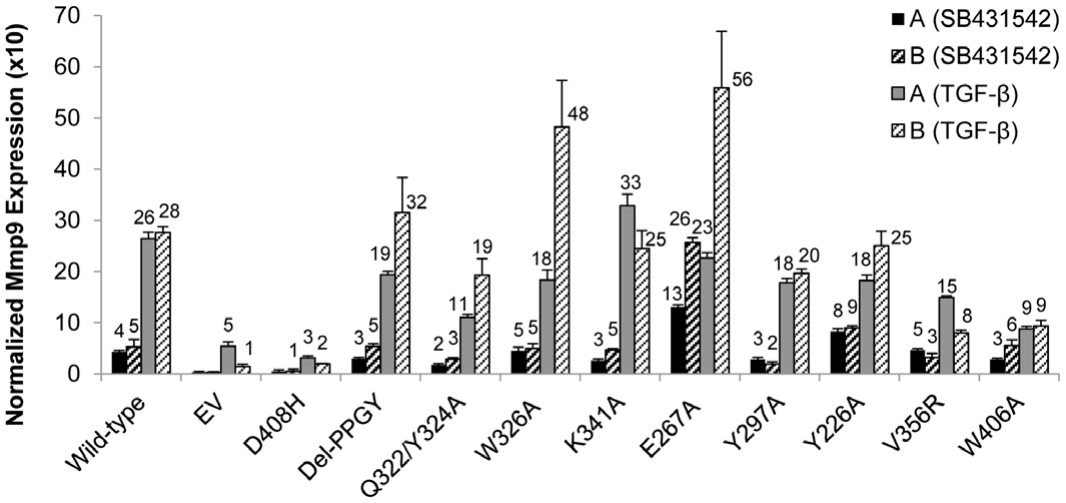
TGF-β induced Mmp9 expression levels are increased by exogenous wild-type Smad3 and some Smad3 variants in C2C12 cells. RNA was isolated from cell lysates prepared from two independent sets (A and B) of C2C12 cell populations independently infected with retrovirus without the myc-Smad3 gene (EV) or retrovirus encoding wild-type or mutant Smad3s. The C2C12 cell populations had been exposed to either 1 µM SB431542 or 100 pM TGF-β for 24 hours before RNA isolation. Mmp9 and beta-actin mRNA expression levels were detected by quantitative RT-PCR in three replicate wells and the MMP9 levels were normalized to the level of beta-actin in each cell lysate. TGF-β increased the expression levels of MMP9 in all of the cell lysates. Expression of wild-type Smad3 increased the basal expression and induced expression levels of Mmp9 five-ten fold over the levels in the cells infected with the EV retrovirus. Expression of Smad3 D408H did not increase the basal expression or induced levels compared to cells infected with the EV retrovirus. Some other Smad3 mutants affected either the basal or induced expression levels (summarized in Table 5). The numerical values indicating the heights of the bars are only shown if the value is >1. The error bars indicate the standard deviations of the three replicate wells of Q-RT-PCR reactions run on each RNA preparation. While the levels of Mmp9 expression differed between the A and B cell populations for some of the mutants, the rank orders across the set of mutants were highly correlated. For SB431542 treated cells, Kendall's rank correlation coefficient (tau) for the A and B cell populations was 0.58 (P = 0.006), and that for TGF-beta treated cells was 0.61 (P = 0.006).

The effect of the Smad3 mutations on the TGF-β-induced expression of six of these genes in C2C12 cells was examined by quantitative RT-PCR (Q-RT-PCR). Expression levels of each gene were normalized to beta-Actin mRNA and compared to levels in C2C12 cells infected with either retrovirus that did not encode Smad3 (Empty vector, EV) or retrovirus expressing the myc-tagged wild-type Smad3. Over-expression of the wild-type Smad3 in the C2C12 cells increased the basal expression levels of the genes even in the absence of TGF-β, but all of the genes exhibited further increases upon exposure to TGF-β for 24 hours. For example, the wild-type Smad3 expression in two independent replicates of infected C2C12 cells increased the basal and TGF-β-induced expression of Mmp9 over cells infected with the control EV retrovirus (Figure 5). In contrast, C2C12 cells expressing the D408H variant of Smad3, which had reduced binding to Smad4 (Table 2) and reduced rescue of the TGF-β-induced reporter gene in JEG-3 cells (Figure 2), had basal and induced levels similar to the EV control cells (Figure 5), even though Smad3 D408H was expressed at about two-fold higher levels than the wild-type myc-Smad3 protein (Figure 3). Although the replicate cell populations expressing the Smad3 mutants varied in their Mmp9 expression relative to beta-actin by two-fold or more in some cases, the trends were consistent between the replicates. The Smad3 mutants Del-PPGY, W326A, and K341A had basal and induced levels similar to wild-type Smad3, whereas V356R and W406A had a reduction in the TGF-β-induced level of Mmp9 (Figure 5). Smad3 E267A and Y226A primarily induced elevated basal levels of Mmp9 (Figure 5). The corresponding analyses for the other five genes, Il11, Tnfaip6, Fermt1, Olfm2 and Wnt11, are shown in the Figure S1A–E.

**Figure 6:**
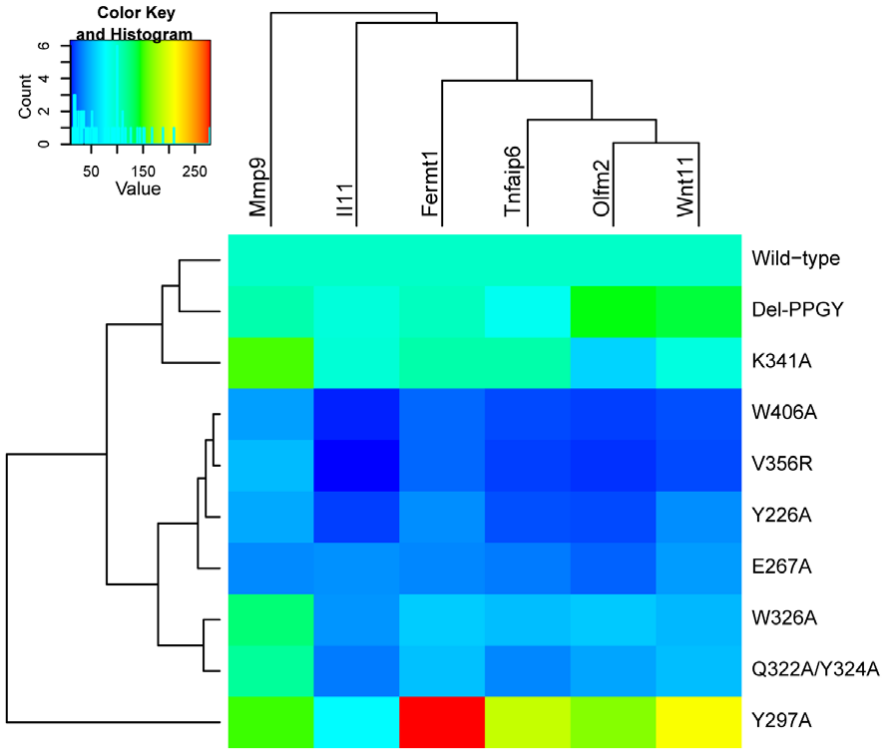
Heat map of gene activation in wild-type and mutant Smad3-expressing C2C12 cells. Expression levels are shown as a percentage of wild-type expression after averaging duplicates. Genes and cells are ordered according to average-linkage hierarchical clustering, as implemented in the heatmap.2 function in the gplots R package [98].

To compare how each mutation affected the TGF-β inducibility of the six genes, the fold increase over basal expression induced by TGF-β treatment for 24 hours was calculated and normalized as the percent of the fold induction mediated by wild-type Smad3 (Table 5). This data was subjected to a cluster analysis (Figure 6). The PPGY deletion and Smad3 K341A variants were similar to wild-type Smad3 in mediating TGF-β inducibility except for the reduced TGF-β inducibility of Olfm2 with Smad3 K341A. Smad3 W406A, V356R, E267A and Y226A clustered together because they reduced TGF-β inducibility between 10–50% of that mediated by wild-type Smad3. However, the gene expression data in Figure 5 and Figure S1 reveal that the reduced TGF-β inducibility occurs by different mechanisms, with V356R and W406A reducing the TGF-β-induced levels and E267A and Y226A increasing the basal levels of gene expression. Note that W406 and V356 are located in the same region of the hydrophobic corridor on Smad3, whereas E267 and Y226 are located in a different region on the surface of Smad3 (Figure 1B). The mutations in the portion of the hydrophobic corridor near the alpha-helix 2, W326A, Q322A/Y324A, also clustered as having intermediate reductions in the levels of expression for the six genes examined with minimal effects on Mmp9 (Figure 6; Table 5). The Y297A mutation was unique in the cluster analysis; it enhanced the TGF-β inducibility of five of the six genes because it exhibited lower basal levels of gene expression than wild-type Smad3.

**Table 5:** Mutations on Smad3 have differential effects on the TGF-β inducibility of six endogenous genes in C2C12 myoblasts.

Smad3 Mutants	Percent of the TGF-β inducibility provided by wild-type Smad3					
	Mmp9	Il-11	Tnfaip6	Fermt1	Olfm2	Wnt11
Wild-type (A)	100 (14)	100 (18)	100 (25)	100 (16)	100 (29)	100 (13)
Wild-type (B)	100 (27)	100 (28)	100 (25)	100 (38)	100 (59)	100 (31)
Del-PPGY (A)	106 (14)	55 (8)	82 (30)	114 (23)	144 (95*)	163 (40*)
Del-PPGY (B)	113 (29)	131 (41*)	84 (24)	93 (27)	145 (65*)	115 (39*)
Q322A/Y324A (A)	102 (19)	24 (4)	39 (11)	71 (24)	44 (11)	57 (11)
Q322A/Y324A (B)	130 (31)	37 (10)	29 (19)	35 (13)	43 (19)	46 (18)
W326A (A)	66 (14)	20 (3)	54 (11)	42 (5)	72 (23)	56 (7)
W326A (B)	186 (68)	56 (13)	49 (28)	72 (24)	40 (18)	42 (11)
K341A (A)	202 (38)	80 (14)	104 (25)	137 (70*)	63 (34*)	105 (21)
K341A (B)	103 (25)	109 (23)	117 (27)	85 (59*)	56 (45*)	77 (28)
E267A (A)	27 (3)	20 (3)	25 (5)	32 (5)	29 (6)	46 (9)
E267A (B)	43 (11)	53 (14)	36 (19)	36 (14)	19 (8)	33 (9)
Y297A (A)	103 (19)	80 (14)	199 (78*)	245 (53*)	130 (34*)	266 (114*)
Y297A (B)	198 (69*)	73 (39*)	178 (42*)	314 (127*)	204 (226*)	154 (50*)
Y226A (A)	35 (5)	13 (2)	18 (4)	31 (6)	16 (4)	33 (5)
Y226A (B)	54 (12)	21 (5)	21 (5)	41 (14)	22 (9)	39 (10)
V356R (A)	52 (6)	10 (1)	16 (3)	29 (8)	17 (5)	20 (3)
V356R (B)	49 (15)	10 (2)	17 (4)	22 (10)	13 (11)	17 (5)
W406A (A)	50 (8)	12 (2)	19 (4)	26 (5)	17 (5)	22 (4)
W406A (B)	33 (10)	13 (3)	19 (4)	24 (9)	16 (9)	18 (4)

Populations of C2C12 cells from two independent infections (A and B) with retroviruses expressing wild-type or mutant Smad3s were treated with 100 pM TGF-β1 (induced) or the TGF-β-receptor kinase inhibitor SB431542 (basal) for 24 hours. Quantitative-RT-PCR was used to quantify the levels of the six indicated gene products and these were normalized to the level of beta-actin mRNA in each lysate. The normalized levels for both the basal and induced cell lysates are shown in Figure 5 and Figure S1. TGF-β inducibility, or fold increase over basal level, was calculated as the ratio of the TGF-β-induced level divided by the basal level of expression. In the C2C12 cells infected with retrovirus expressing wild-type Smad3 (both the A and B infections), the fold increases over basal induced by TGF-β were: MMP9 6±1; Il-11 31±5; Tnfaip6 11±2; Fermt1 8±2; Olfm2 31±10; Wnt11 12±2. The TGF-β inducibility provided by the mutant Smad3 proteins was normalized to the TGF-β inducibility provided by wild-type Smad3 in the infected cells which was set to 100%. The standard deviations are indicated in parenthesis.

*Variance in the very low basal expression levels were the primary contributors to the large standard deviation.

## Discussion

We have reported on some protein binding defects and gene expression changes caused by the mutation of specific amino acids on the surface of Smad3. As these amino acids are conserved in Smad2, the corresponding mutations in Smad2 might also affect Smad2 function, but Smad2 was not examined in these studies. The protein ligands used to query the binding properties of the Smad3 variants were a small subset of the identified Smad3-interacting proteins. They were used to reveal whether a mutation in a putative hot-spot on Smad3 reduced the binding to one or more proteins and, importantly, retained the binding to other Smad-binding partners, i.e., that each Smad3 mutation only affected binding to a portion of this test set of protein and peptide ligands. Although the test set was meant to represent the larger (>50) group of known Smad3-interacting proteins and was large enough to characterize selective protein-binding deficits in the variants, the test set of proteins and peptides cannot reveal the full extent of the protein-binding defects or which other Smad3 protein-protein interactions may be reduced by a specific mutation. As such, the effects of the mutations on the transcriptional functions of Smad3 are very likely mediated by other Smad3-interacting proteins that were not in the test panel. Therefore, one should not conclude that any of the specific protein ligands used in the reported binding studies are directly implicated in mediating the reported gene expression changes. One exception to this caveat may be the mutations that reduce binding to Smad4. Given that the canonical function of phosphorylated Smad3 is to form a heterotrimer with Smad4 that accumulates in the nucleus, reduced Smad4 binding is likely to be in part responsible for the reduced activity of these mutations in reconstituting Smad3-dependent reporter gene activity in JEG-3 cells.

The protein binding properties of the Smad3 variants were quantified in a modified version of the LUMIER assay [48]. Proteins were co-expressed in HEK-293 cells with an activated Alk5 receptor to constitutively phosphorylate Smad3. All of the previously described Smad3 mutations exhibited the same defects in protein binding in this assay as had been initially reported using other assay methods. By using a larger panel of Smad3-binding proteins and peptides, we identified additional binding interactions that were impaired by each mutation and also demonstrated that other binding interactions were not affected under the conditions of the assay. In the cases where both a Smad binding domain and the corresponding full length protein were tested, the full length protein always provided more binding to the Smad3 variant. The increased binding by the full length protein may indicate that the Smad binding domain is in a more optimal conformation in the native protein than on the Trx scaffold or that the full length protein has other motifs that contribute to the binding in addition to the motif displayed on the Trx scaffold. In either case, full-length Smad-binding proteins will be a better indicator in future studies of the consequences of a hot-spot mutation in cells.

The binding assays revealed several examples where the amount of binding between a Smad3 variant and a Smad-binding protein was greater than with the wild-type Smad3 protein. For example, more Renilla-Smad3 E267A was pulled-down with Smad4, Smad3 and the Smad-binding domains of Smurf2 and Ski. Greater amounts of Renilla luciferase Smad3 V224A were pulled-down with SkiW274E and the Smad-binding domains from Smurf2, Ski and SIP1, but not by Smad3 or Smad4. The Smurf2 Smad binding domain pulled down more of nine different Renilla-Smad3 variants in Table 3. Previous studies have reported a propensity for mutant Smad proteins to undergo degradation, which would be consistent with increased interactions with an E3 ubiquitin ligase such as Smurf2 [66]. The molecular mechanism(s) for the increased binding in the present studies are not known. The mutations may enhance the binding affinity between Smad3 and a Smad-binding protein either by improving the binding interface directly or by altering the conformation of Smad3 to improve the binding interface. An alternative explanation is that these mutations reduce the amount of some Smad3-binding proteins interacting with Smad3, thereby making more Smad3 available to interact with other Smad3-binding proteins. Independent of the mechanism, the results indicate that the functional consequences of perturbing a protein-binding hot-spot on a hub protein by mutation or with a small molecule ligand or drug may be the result of both decreased interactions with some protein ligands and also increased interactions with other protein ligands. For example, the Smad3 E267A protein exhibited increased binding to several protein ligands and mediated increased basal levels of gene expression and wild-type (or greater than wild-type) TGF-β-induced levels of gene expression on several of the genes examined in C2C12 cells. This high basal activity resulted in a lower fold-induction by TGF-β, as indicated by the lower TGF-β inducibility in Table 5 and Figure 6. The increase in basal gene expression by Smad3 E267A was not due to it being expressed at higher levels of protein expression than the exogenous wild-type Smad3 or the other variants (Figure 3). In future studies on the functional deficits of Smad3 variants it will be important to examine both the loss of binding partners and the increase of other Smad3-binding proteins in the regulatory complex relative to the components of the wild-type Smad3 regulatory complex.

Although only a small panel of Smad3-reponsive genes was examined, there was some evidence for different hot-spot mutations causing distinct effects on gene expression. For example, the Y297A mutation, decreased binding to SARA and Ski (Table 3) and MEF2C and the Smad binding domain from FoxO3 (Table S1) but provided 100% reconstitution of the Smad3-dependent reporter gene activation in JEG-3 cells (Figure 2). However, Smad3 Y297A also had the unique property among the mutations examined of enhancing the TGF-β induciblity of five of the endogenous genes tested in C2C12 cells. Overexpression of wild-type Smad3 is well-known to induce gene expression changes in the absence of TGF-β, but the Smad3 Y297A variant induced lower levels of expression in the absence of TGF-β than did wild-type Smad3, resulting in a greater fold induction when TGF-β was added.

Another example for a selective effect was observed with Smad3 K341A. The K341A mutation decreased binding to SARA and Ski (Table 3) and the Smad binding peptide from CBP (Table S1) and provided less than 50% reconstitution of the Smad3-dependent reporter gene activation in JEG-3 cells (Figure 2), but it mediated TGF-β inducibility similar to wild-type Smad3 for five of six endogenous genes tested. Interestingly, it was compromised by almost 50% for mediating TGF-β activation of Olfm2. Examination of a larger panel of Smad3 responsive genes and expression of the Smad3 K341A protein in an otherwise Smad3-deficient cell line may identify genes with an even greater dependence than Olfm2 on K341 in Smad3.

Several genes were identified from a gene array analysis of mRNAs from the TGF-β-induced C2C12 cells over-expressing wild-type Smad3 versus TGF-β-induced C2C12 cells infected with the control retrovirus that did not encode Smad3 (empty vector, EV). RT-PCR analysis confirmed the increased level of expression for thirteen genes and a reduced level of expression for three genes after 24 hours of TGF-β treatment; in most cases the responses were amplified by the overexpression of wild-type Smad3. Although we did not select for any particular functionality, most of the genes with increased expression levels encode proteins with roles in cell signaling, cell adhesion or extracellular matrix modifications. Several of the genes including Pai1, Mmp9 and Il11 have been identified as TGF-β-responsive genes in many other studies and cell-types. For example, Pai1 is induced by TGF-β in muscle satellite cells [67] and MMP9 is an important Smad3 target in the process of cancer cell invasion [28]. Regulation of Il11 by TGF-β signaling is important for metastasis to bone of breast and melanoma cancer cells [68], [69], [70]. Pai1 and Cst6 were identified as direct targets for Smad3 regulation in a genome wide chromatin immunoprecipitation study [71]. Cst6 encodes cystatin M/E, an inhibitor of the cathepsin L cysteine protease, which has been implicated in epidermal differentiation and tumor suppression [72]. Methylation or loss of cystatin M/E expression has been reported in breast [73] and prostate cancers [74]. To the best of our knowledge Cst6 expression has not previously been reported in myoblasts.

The two other genes whose expression levels were reduced in the C2C12 cells were Myl1 and Sarcoglycan, Sgcg. Myl1 has been associated with muscle cell differentiation and knock-down of its expression enhanced myoblast proliferation [75]. Sgcg mutations cause limb-girdle muscular dystrophy and Smad3 has been implicated in preventing inappropriate activation of Sgcg gene expression during myogenic differentiation [76]. The reduced levels of Myl1 and Sgcg are consistent with the inhibition of muscle differentiation by TGF-β and Smad3. One of the earliest cellular responses reported for TGF-β was inhibition of myoblast differentiation in culture [77], [78]. Smad3 binds directly to the MyoD bHLH domain to block MyoD/E protein dimerization and DNA binding [60] and to the myogenic transcription factor MEF2 to prevent muscle-specific gene expression [61]. Persistent TGF-β expression contributes to the muscle fibrosis and loss of muscle function in neuromuscular diseases such as Duchenne muscular dystrophy [79], [80].

Fermt1 encodes Kindlin-1, a focal adhesion protein involved in cytoskeletal interactions and implicated in integrin beta-1 activation [81], [82]. Loss-of-function of Fermt1 is associated with Kindler syndrome, an autosomal recessive disorder characterized by skin atrophy and blistering [83]. Kindlin-1 is a member of a family of proteins that are key regulators of cell-matrix interactions [84]. Tnfaip6 or TGS-6 (tumor necrosis factor, alpha-induced protein 6) encodes a hyaladherin, a hyaluronan-binding protein, and is involved in extracellular matrix stability and cell migration [85]. TGS-6 expression is induced by TGF-β in dermal fibroblasts and is a required mediator of the TGF-β induced fibroblast-myofibroblast transition in wound healing [86], [87]. Olfm2 or olfactomedin 2 is a secreted glycoprotein belonging to a family of related olfactomedins [88]. We are not aware of previous reports of a role for olfactomedins in muscle or myoblasts. A related family member, Olfm1, has been reported as a modulator of Wnt signaling in zebrafish [89]. The Wnt family member that was induced in the studies reported here, Wnt11, has been implicated in fostering cell adhesion of cardiomyocytes essential for proper heart development [90].

Understanding the multiple functions of hub proteins requires new approaches. Knockdown approaches or knock-out mutations do not allow analysis of the distinct roles of a hub protein. Mutations in protein-binding hot-spots have the potential to only perturb a subset of the total binding interactions, providing an opportunity to learn the biological significance of those interactions. This approach is analogous to the generation of an allelic series of mutations in classical genetics for the purpose of revealing the different developmental roles of a specific gene. Future studies will benefit from a more comprehensive analysis of the gene expression changes caused by each hot-spot mutation, from analysis of the biological consequences on cellular phenotypes such as proliferation, differentiation, or pathological functions and from characterization of the changes in the composition of the protein complexes formed on the hub protein in the same cell-type and cell context being studied for phenotypic changes.

## Materials and Methods

### Cell lines and cDNA clones

JEG-3, HEK-293, and C2C12, cells were obtained from the American Type Culture Collection (HTB-36, CRL-1573, CRL-1772 and, respectively; Manassas, VA) and maintained at 37°C, 5% CO_2_. JEG-3 cells were maintained in MEM (Modified Essential Medium), and supplemented with 10% fetal bovine serum (FBS) (Gibco, Invitrogen, Carlsbad, CA). HEK-293 cells were grown in DMEM (Dulbecco's Modified Eagle Medium, Gibco, Invitrogen) supplemented with 5% FBS and the murine C2C12 cells were grown in DMEM supplemented with 10% FBS.

The following plasmids were generously provided by the indicated individuals: pcDNA3-ALK5, Carl-Henrik Heldin (Ludwig Institute for Cancer Research, Uppsala, Sweden); pME-Flag-ATF3, Shigetaka Kitajima (Tokyo Medical and Dental University Tokyo, Japan); pCS2-Flag-mAxin, Xi He (Children's Hospital Boston, Boston MA); pCMV5-Flag-FoxO3, Smad4, Smad3, Smad3-V356R, Smad3-Y297A, Smad3-K341A, Smad3-K340A, Smad3-C338A, Joan Massagué (Sloan Kettering Cancer Center, New York, NY); pCMV-Flag-hHNF4alpha, Paul Kuo (Duke University Medical Center Durham, NC); HA-SRF, John Lough (Medical College of Wisconsin, Milwaukee, WI); pcDNA3-Flag-hMEF2C(1-302), Zhenguo Wu (Hong Kong University of Science & Technology, Clear Water Bay, NT, Hong Kong); CMV10-3XFlag-Myo-D, Jin Cheng (H. Lee Moffitt Cancer Center and Research Insitute, Tampa, FL); pcDNA3-Flag-Smad3(STSVL/NKN), Kohei Miyazono (University of Tokyo, Tokyo, Japan); SBE12-luciferase, Peter ten Dijke (Leiden University Medical Center, Leiden, Netherlands); pCMV5b-Flag-SkiW274E, Kunxin Luo (University of California, Berkeley, CA); pACT2-SIP1-315-487, Danny Huylebroeck (Flanders Institute for Biotechnology, Leuven, Belgium), pCMV5b-Flag-SARA (Addgene plasmid 11738) and pCMV5-hRLuc-Smad3, Jeffrey Wrana (Samuel Lunenfeld Research Institute, Mount Sinai Hospital,Toronto, Canada).

The following Smad binding domains were isolated by PCR and cloned into the pCI-3xFLAG-Trx plasmid using a unique RsrII site: CBP amino acids 1940–1998 [31], [49], xFoxH1 amino acids 293–317 [44], [49], SRF amino acids 132–223 [91], ATF3 amino acids 61–111 [92], FoxO3 amino acids 153–249 [93], SARA amino acids 665–721 [32], [51], Ski amino acids 2–56 [22], and SIP1 amino acids 315–487 [94].

### Smad3 Mutants

Mutations were created using site directed mutagenesis [95]. Smad3 mutations were created in the Renilla luciferase pCMV5-hRLuc Smad3 using PfuUltraII Fusion hot-start DNA polymerase (Stratagene, La Jolla, CA). Mutations were verified by sequence analysis of the entire expression construct at the University of Wisconsin Biotechnology Center. Mutated Smad3 genes were digested with restriction endonucleases and inserted into the pCMV5 3xmyc-Smad3 vector or the pCMMV-IRES-GFP 3xmyc vector.

### Retrovirus Production and Infection

To generate retroviruses, HEK-293 cells were plated at 1×10^6^ cells in a 10 cm dish and transfected 24 hours later with a pCMMV-IRES-GFPvector [96] containing either no insert (empty vector, EV), wild-type Smad3 (WTSmad3), or mutant Smad3 (5 µg DNA per plate) as well as three plasmids encoding vesicular stomatitis virus G protein (VSVG) (0.5 µg DNA per plate), gag/pol elements (1.5 µg DNA per plate), and NFκB (0.5 µ per plate) using the *Trans*IT-LT1 Transfection Reagent (Mirus Bio LLC, Madison, WI) at a transfection reagent to DNA ratio of 3∶1. Cells were incubated overnight at 37°C, 5% CO_2_. Media with transfection reagent was removed and replaced with DMEM containing 5% FBS with 50 mM HEPES. Supernatants were collected at 48 and 72 hours. Supernatants were centrifuged at 1500 rpm for 10 minutes at 4°C and filtered through 0.2 µm filters. Filtered supernatants were centrifuged at 20,000 rpm for two hours at 4°C. Virus pellets were resuspended in DMEM containing 5% FBS with 50 mM HEPES and stored at −80°C.

For infection, C2C12 cells were plated at 1×10^5^ cells in a 6 cm dish and infected the following day with a total volume of 1.2 ml of 10 µg/ml polybrene and 40–175 µl of concentrated retroviruses in DMEM containing 10% FBS at 4°C with rocking for one hour. Then, 3 ml of DMEM containing 10% FBS was added to each plate and incubated overnight at 37°C, 5% CO_2_. Cells were allowed to propagate for no longer than one week before experimental analysis. The infection process was repeated twice for each mutant.

### Luminescence-based mammalian interactome mapping (LUMIER) assay

This is a modification of the assay originally described by Dr. Jeffery Wrana and colleagues [48]. HEK-293 cells were seeded in 12 well plates and transfected with pCMV-hRLuc Smad3 wild-type or variants (0.2 ug/well), Flag-tagged Smad binding partner (see cell lines and cDNA clones section) (0.2 ug/well), and a plasmid expressing the constitutively active form of the type I receptor, Alk5 (0.15 ug/well) using *Trans*IT-LT1 transfection reagent (Mirus Bio LLC). Cells were incubated for 24 hours before harvesting. White 96-well anti-Flag coated plates were prepared by binding 0.008 µg anti-Flag antibody (A8592, Sigma) to Reacti-bind protein G coupled plates (Pierce, Thermo, Rockford IL) at 4°C for one hour, according to the plate manufacturer's instructions. Transfected cells were lysed on ice for 15 minutes in 200 µl TNE lysis buffer: 10 mM Tris-HCl pH 7.8, 150 mM NaCl, 1 mM EDTA, 1% Igepal CA-630 (Sigma-Aldrich, St. Louis, MO), and Roche complete mini protease inhibitor (Roche, Indianapolis, IN). The debris was pelleted out of the lysates by centrifugation at 14,000 rpm for 12 minutes at 4°C and the cleared lysates were added to the anti-Flag coated plates. The plate was incubated for two hours at 4°C with gentle vortexing and washed with 0.05% Tween-20 Tris-buffered saline four times. Renilla luciferase activity was measured with the Dual-Glo Assay system (Promega,Madison, WI) as follows: 20 µl of serum free-Dulbecco's Modified Eagle Medium (DMEM) was added to each well of the plate followed by 20 µl of Dual-Glo Luciferase Buffer (this buffer is used without firefly-luciferase substrate added for the purpose of maintaining proper buffer composition for the Renilla-Luciferase substrate). Immediately prior to analyzing the plate, 20 µl of Dual-Glo Stop-N-Glo buffer containing Renilla-Luciferase substrate was added to each well. The plate was analyzed on a PerkinElmer Wallac 1420 Victor Plate Reader which measured relative light units in each well. As a transfection control, 5 µl of debris-free lysate was analyzed for the total Renilla luciferase activity as described above, using 5 µl each of serum free-DMEM, Dual-Glo Luciferase Buffer and Dual-Glo Stop-N-Glo/Renilla substrate. A pCI-3xFlag-TrxGA construct, which consists of a tandem repeat of glycine and alanine and does not bind to Smad3 [49], was used as a control in every experiment to determine the level of background non-specific luciferase binding. The background signal obtained with TrxGA was subtracted from each sample and the data for each Smad3 mutant was normalized to wild-type Smad3 Renilla levels and shown as a percent.

### Reporter gene assay

For rescue experiments in the JEG-3 cell line, 3×10^5^ cells per well were seeded in 24-well plates and allowed to adhere overnight. Cells were transfected with appropriate plasmids using *Trans*IT-LT1 transfection reagent (Mirus Bio) at a transfection reagent to DNA ratio of 3∶1. The transfected plasmids included: SBE12-luc reporter gene [97] (10 ng per well), a reporter plasmid encoding CMV-β-galactosidase (β-gal) (10 ng per well), pCMV5 Renilla luciferase Smad3 (20 ng per well). An empty vector pCI plasmid was used to normalize the amount of DNA transfected to a total of 100 ng per well. Eight to 12 hours after transfection, media was removed and replaced with 100 pM TGF-β (R & D Systems, Minneapolis, MN). Twelve hours after TGF-β treatment, cells were washed with phosphate buffered saline (PBS) and lysed with 75 µl of lysis buffer (Galacto-Star kit, Applied Biosystems, Invitrogen, Carlsbad, CA) for 15 minutes. β-galactosidase activity was measured using 5 µl of cell lysate plus 100 µl of Galacto-Star β-gal substrate. Luciferase activity was measured with 50 µl of cell lysate plus 50 µl of Bright-Glo luciferase substrate (Promega) and Renilla-luciferase was measured with 5 µl of cell lysate (Dual-Glo Luciferase kit, Promega). β-galactosidase values were used for normalization of the luciferase value. Data in each experiment are shown as the mean ± standard deviation.

### Quantitative Western for Myc-Smad3 expression

Stable C2C12 cells were treated with 1 µM SB431542 (Tocris Bioscience, Ellisville, MO) or 100 pM TGF-β for 24 hours prior to lysis in TNE lysis buffer, and Roche complete mini protease inhibitor at 4°C. Lysates were cleared of debris by centrifugation at 14,000 rpm for 12 minutes at 4°C and proteins were separated by SDS-PAGE in 4–20% gradient midi gels (NuSep, Lane Cove, NSW Austrailia) and transferred to low background fluorescence membrane, Immobilon F PVDF (Millipore, Billerica, MA). A 1∶2 solution of PBS and Seablock (Pierce, Thermo) was used for blocking and antibody dilution. Primary antibodies, 0.4 µg/ml mouse monoclonal IgG_1_ cMyc 9E10 (Santa Cruz SC-40, Santa Cruz, CA) and 1.2 µg/ml rabbit polyclonal to beta Tubulin (Abcam ab6064) were applied overnight at 4°C. LI-COR (Lincoln, NE) IRDye secondary antibodies were subsequently applied; 67 ng/ml Goat anti-Mouse IRDye 800 CW (LI-COR, 827-08364) and 67 ng/ml Goat anti-Rabbit IRDye 680 (LI-COR, 926-32221) for one hour at room temperature. Blots were imaged on the LI-COR Odyssey Infrared Imaging System. Myc-Smad3 protein expression was quantified relative to beta-Tubulin, as follows: a two-fold dilution series of wild-type Smad3 cell lysate, from 0.31 µg to 5.0 µg, and 2.5 µg mutant Smad3 cell lysates were compared. The Odyssey 3.0 analysis software was used to estimate the integrated intensity of each myc-Smad3 and beta-Tubulin band. Standard curves of both myc-Smad3 and beta-Tublin were generated from the two-fold dilution series of wild-type Smad3. The standard curves were used to convert each integrated intensity to a total protein value and the total protein value for each myc-Smad3 was divided by the total protein value for the beta-Tubulin from the same sample to normalize for differences in sample loading.

### RNA isolation, RT-PCR and Q-RT-PCR Methods

RNA was isolated from C2C12 cells plated in 10 cm dishes treated with either 1 µM SB431542 or 100 pM TGF-β using the RNeasy Plus kit (Qiagen, Valencia, CA). Quantification of RNA was done using a Nanodrop spectrometer and 1 µg of RNA was used for a single cDNA reaction (Superscript II, Invitrogen) along with oligodT primers following the manufacturer's protocol.

The expression of the following genes were quantified by RT-PCR or Q-RT-PCR: Beta-Actin (NM_007393); Pai1, Serpine 1 (NM_008871); Cst6, cystatin E/M (NM_028623); Mmp9, matrix metallopeptidase 9 (NM_013599); Il11, interleukin 11 (NM_008350); Tnfaip6, tumor necrosis factor alpha induced protein 6 (NM_009398); Fermt1, fermitin family homolog 1 (NM_198029); Oflm1, olfactomedin 1 (NM_173777); Wnt11, wingless-related MMTV integration site 11 (NM_009519); Adamts4, a disintegrin-like and metallopeptidase with thrombospondin type 1 motif 4 (NM_172845); Clu, clusterin (NM_013492); Edn1, Endothelin 1 (NM_010104); Gfpt2, glutamine fructose-6-phosphate transaminase 2 (NM_013529); Myl1, myosin light polypeptide 1 (NM_021285); Npy, neuropeptide Y (NM_023456); Pdgfb, platelet derived growth factor, B polypeptide (NM_011057); Sgcg, sarcoglycan, gamma (NM_011892) (see Table S3 for primer and probe sequences).

RT-PCR was performed using 1 µl of undiluted cDNA, 0.2 µM of forward and reverse primers, and Go Taq Green Master Mix 2× (Promega) at a final concentration of 1×. The RT-PCR was run in a thermocycler with the following program: 95°C for 3 minutes; 30 or 35 cycles of 95°C for 30 seconds, 58°C for 45 seconds, 72°C for 1 minute; 72°C for 8 minutes. RT-PCR products were run on a 2% agarose gel with ethidium bromide and imaged using a gel documentation system (BioRad, Hercules, CA).

For Q-RT-PCR reactions, cDNA was diluted 1∶4 with water and reactions were set up in triplicate. The Q-RT-PCR was conducted with 500 nM of forward and reverse primers (UW Biotechnology Center) and 200 nM FAM-labeled probes with an internal ZEN quencher and 3′ Iowa Black quencher (PrimeTime QPCR probes, Integrated DNA Technologies, Coralville, CA) in a 20 µl reaction set up in 384-well plates (Applied Biosystems). AmpliTaq Gold PCR master mix (Applied Biosystems) was used at 1× in all experiments. All experiments were run on the ABI 7900HT using the standard thermo cycling protocol. Data were analyzed based on a standard curve which was performed for each gene in every experiment. A pool of cDNA from empty vector and wild-type Smad3- expressing cells treated with either 1 µM SB431542 or 100 pM TGF-β for 24 hours were used to generate the standard curves. Relative quantities from 1-0.00521 (a 2-fold dilution series from 1-1∶64 plus 1∶192) was used to generate the standard curve. Controls with no cDNA template added were conducted for each gene in every experiment. The SDS 2.4 software (Applied Biosystems) was used to analyze all PCR experiments by generating a standard curve and estimating the relative quantity of each unknown sample. The slope of the standard curve for all experiments was ≥−4. Data were normalized to the relative expression level of beta-actin, also generated by a standard curve. To calculate the fold induction, the quantity of each sample after TGF-β treatment was divided by the quantity of each sample after SB431542 treatment. This was then normalized to the wild-type Smad3 fold induction as a percentage in Table 5.

### Mouse Affymetrix Gene Expression Array Methods

Two biological replicates of RNA samples from each wild-type Smad3-expressing C2C12 or control (Empty vector) C2C12 cells treated for 24 hours with 100 pM TGF-β were submitted to the UW Genome Expression Center for analysis of gene expression using Affymetrix (Santa Clara, CA) Mouse Gene ST 1.0 microarrays. To identify genes with altered expression between duplicated empty vector controls and wild-type Smad3, we first pre-processed the four arrays using robust multiarray averaging as implemented in the R software package ‘xps’, version 1.10.2 (http://www.bioconductor.org/packages/release/bioc/html/xps.html). This preprocessing corrects for background noise and array effects, and aggregates probe data to 28,836 genes. Duplicates were averaged on the base-2 logarithm scale, and then we took differences between the averages to obtain logarithm-scale fold changes. Genes were selected which showed at least a two-fold change (raw scale) between wild-type and empty vector expressing cells. Due to the limited sample size, we did not apply any statistical tests to estimate false-discovery rate. This resulted in 79 genes that were at least two-fold higher in the wild-type Smad3-expressing C2C12 cells compared to empty vector cells and 25 genes that were at least two-fold lower in the wild-type Smad3-expressing cells than the in the empty vector cells. Candidate genes were selected and expression differences were confirmed using RT-PCR. All microarray data is MIAME compliant and the raw data has been deposited in and is available at GEO (http://www.ncbi.nlm.nih.gov/geo/).

### Statistical Analysis

The calculation of standard deviation of the ratios, multiexperiment permutation test, the Spearman's rank correlation coefficients and the Kendall's rank correlation coefficients was done using the Mstat program (http://www.mcardle.wisc.edu/mstat/). Cluster analysis of gene expression data was performed according to average-linkage hierarchical clustering, as implemented in the heatmap.2 function in the gplots R package [98].

## Supporting Information

Figure S1 **TGF-β-induced expression levels of five endogenous genes in C2C12 cells are increased by exogenous wild-type Smad3 but different mutant Smad3s exhibit a variety of alterations in the basal and TGF-β-induced expression levels.** RNA was isolated from cell lysates prepared from two independent sets (A and B) of C2C12 cell populations independently infected with retrovirus without the myc-Smad3 gene (EV) or retrovirus encoding wild-type or mutant Smad3s. The C2C12 cell populations had been exposed to either 1 µM SB431542 or 100 pM TGFβ for 24 hours before RNA isolation. Expression levels of beta-actin mRNA and (A) Il11, (B) Tnfaip6, (C) Fermt1, (D) Olfm2 or (E) Wnt11 were detected by quantitative RT-PCR in three replicate wells. The expression levels of the five genes were normalized to the level of beta actin in each cell lysate. Cells infected with retrovirus expressing wild-type Smad3 had higher basal and TGF-β-induced expression levels for all five genes (panels A–E) than cells infected with the EV retrovirus. In contrast, cells infected with retrovirus expressing Smad3 D408H had similar levels of expression to the cells infected with the EV retrovirus. Other Smad3 mutants affected either the basal or induced expression levels or both. The numerical values indicating the heights of the bars are only shown if the value is >1. The error bars indicate the standard deviations of the three replicate wells of Q-RT-PCR reactions run on each RNA preparation.
(TIF)

Table S1 **Mutations on Smad3 MH2 have differential effects on interactions with Smad-binding proteins.** Binding between wild-type or mutant Renilla-Smad3 fusion proteins and nine different Flag epitope-tagged Smad-binding proteins was quantified by pull-down of protein complexes from cell lysates and detection of the Renilla luciferase activity per well. The Renilla luciferase counts were normalized to the number of counts recovered with phosphorylated wild-type Smad3 (100%). The proteins were all co-expressed with the constitutively active Alk5. FL indicates that the full-length protein was used. The Smad3 MH2 domain has been reported to bind directly to CBP [99], xFoxH1 [100], MyoD [60], MEF2C [61], Axin [62], Serum response factor (SRF) [91] and ATF3 [92]. The Smad3 MH1 domain mediates Smad3 binding to Hnf4 [101] and FoxO3 [93]. The amino acids comprising the CBP [31], xFoxH1 [44], [100], SRF [91], ATF3 [92] and FoxO3 [93] Smad3-binding motifs expressed on the Flag-epitope tagged thioredoxin scaffold are indicated below each gene. The reduced binding to the Smad interaction motif from xFoxH1 by mutations in the alpha-helix 2 region of Smad3 (W326A and Q322A/Y324A) is consistent with previous studies mutating the corresponding amino acids in Smad2 [44]. Reduced binding to the Smad interaction motif from CBP by Smad3 W406A also is consistent with a prior study on this protein interaction that used size exclusion chromatography with purified proteins [64]. Standard deviations are indicated in parenthesis. * The Smad3 QPSMT/SE mutant has residues 252–256 and 266–267 changed to the Smad1 equivalent amino acids (STSVL/NKN) [47].
(DOC)

Table S2 **Effects of single alanine mutations in the Smad3 MH2 region implicated in binding to Ski on interactions with Smad3-binding proteins.** Binding between wild-type or mutant Renilla-Smad3 fusion proteins and eight different Flag epitope-tagged Smad-binding proteins was quantified by pull-down of protein complexes from cell lysates and detection of the Renilla luciferase activity per well. The Renilla luciferase counts were normalized to the number of counts recovered with phosphorylated wild-type Smad3 (100%). The proteins were co-expressed with the constitutively active Alk5. FL indicates that the full-length protein was used. The amino acids comprising the SARA [32], Smurf2 [52], Ski [22], and Sip1 [94] Smad3-binding motifs expressed on the Flag-epitope tagged thioredoxin scaffold are indicated below each gene. The reduced binding by Smad3 with mutations in the QPSMT/SE motifs to the Ski Smad-binding domain and full length SkiW274E is consistent with previous studies [47]. The S254A mutation was the only single amino acid mutation that significantly reduced binding to the Ski Smad binding domain. Unexpectedly, E267A enhanced Smad3 interactions with several Smad-binding proteins. Standard deviations are indicated in parenthesis.
(DOC)

Table S3 **Primer and probe sequences used for RT and Q-RT-PCR.** Primer and probe (where applicable) sequences used for RT and Q-RT-PCR. Probes were labeled with 5′ FAM and 3′ Iowa Black with an internal ZEN quencher.
(XLS)
