# Supplementary figures and images for: Mutations in Protein-Binding Hot-Spots on the Hub Protein Smad3 Differentially Affect Its Protein Interactions and Smad3-Regulated Gene Expression
Source: PLoS One. 2011 Sep 19;6(9):e25021. doi: 10.1371/journal.pone.0025021 (PMC3176292; doi:10.1371/journal.pone.0025021)

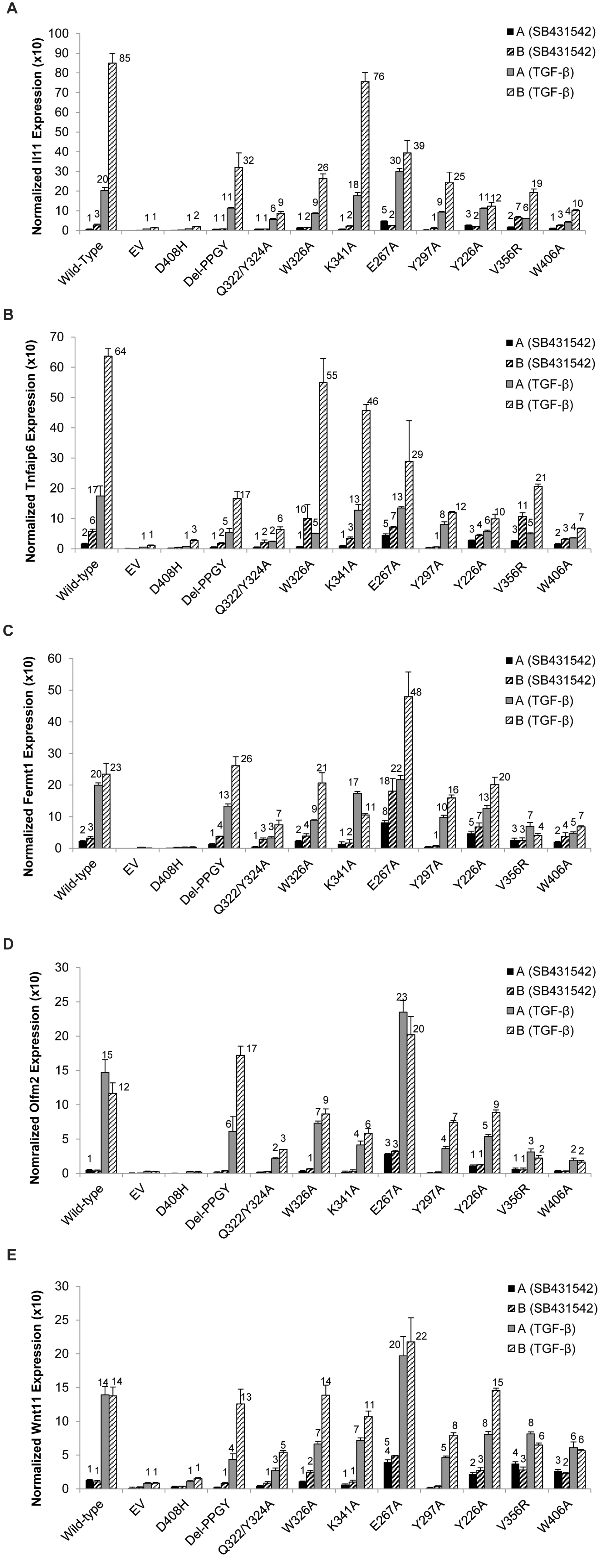

Supplement: Figure S1 — TGF-β-induced expression levels of five endogenous genes in C2C12 cells are increased by exogenous wild-type Smad3 but different mutant Smad3s exhibit a variety of alterations in the basal and TGF-β-induced expression levels. RNA was isolated from cell lysates prepared from two independent sets (A and B) of C2C12 cell populations independently infected with retrovirus without the myc-Smad3 gene (EV) or retrovirus encoding wild-type or mutant Smad3s. The C2C12 cell populations had been exposed to either 1 µM SB431542 or 100 pM TGFβ for 24 hours before RNA isolation. Expression levels of beta-actin mRNA and (A) Il11, (B) Tnfaip6, (C) Fermt1, (D) Olfm2 or (E) Wnt11 were detected by quantitative RT-PCR in three replicate wells. The expression levels of the five genes were normalized to the level of beta actin in each cell lysate. Cells infected with retrovirus expressing wild-type Smad3 had higher basal and TGF-β-induced expression levels for all five genes (panels A–E) than cells infected with the EV retrovirus. In contrast, cells infected with retrovirus expressing Smad3 D408H had similar levels of expression to the cells infected with the EV retrovirus. Other Smad3 mutants affected either the basal or induced expression levels or both. The numerical values indicating the heights of the bars are only shown if the value is >1. The error bars indicate the standard deviations of the three replicate wells of Q-RT-PCR reactions run on each RNA preparation. (TIF) [file pone.0025021.s001.tif]
