# Supplementary material for: Mutations in Protein-Binding Hot-Spots on the Hub Protein Smad3 Differentially Affect Its Protein Interactions and Smad3-Regulated Gene Expression
Source: PLoS One. 2011 Sep 19;6(9):e25021. doi: 10.1371/journal.pone.0025021 (PMC3176292; doi:10.1371/journal.pone.0025021)
Supplement: Table S1 — Mutations on Smad3 MH2 have differential effects on interactions with Smad-binding proteins. Binding between wild-type or mutant Renilla-Smad3 fusion proteins and nine different Flag epitope-tagged Smad-binding proteins was quantified by pull-down of protein complexes from cell lysates and detection of the Renilla luciferase activity per well. The Renilla luciferase counts were normalized to the number of counts recovered with phosphorylated wild-type Smad3 (100%). The proteins were all co-expressed with the constitutively active Alk5. FL indicates that the full-length protein was used. The Smad3 MH2 domain has been reported to bind directly to CBP [99], xFoxH1 [100], MyoD [60], MEF2C [61], Axin [62], Serum response factor (SRF) [91] and ATF3 [92]. The Smad3 MH1 domain mediates Smad3 binding to Hnf4 [101] and FoxO3 [93]. The amino acids comprising the CBP [31], xFoxH1 [44], [100], SRF [91], ATF3 [92] and FoxO3 [93] Smad3-binding motifs expressed on the Flag-epitope tagged thioredoxin scaffold are indicated below each gene. The reduced binding to the Smad interaction motif from xFoxH1 by mutations in the alpha-helix 2 region of Smad3 (W326A and Q322A/Y324A) is consistent with previous studies mutating the corresponding amino acids in Smad2 [44]. Reduced binding to the Smad interaction motif from CBP by Smad3 W406A also is consistent with a prior study on this protein interaction that used size exclusion chromatography with purified proteins [64]. Standard deviations are indicated in parenthesis. * The Smad3 QPSMT/SE mutant has residues 252–256 and 266–267 changed to the Smad1 equivalent amino acids (STSVL/NKN) [47]. (DOC) [file pone.0025021.s002.doc]

**Table S1. Mutations on Smad3 MH2 have differential effects on interactions with Smad-binding proteins.**

| **Smad3 Mutants** | **Percent binding to Smad3 mutants relative to the binding to wild-type Smad3 (100%)** | | | | | | | | |
| --- | --- | --- | --- | --- | --- | --- | --- | --- | --- |
|  | **CBP** | **xFoxH1** | **MyoD** | **MEF2C** | **Axin1** | **SRF** | **ATF3** | **FoxO3** | **HNF4** |
|  | **1940-1988** | **293-317** | **FL** | **1-302** | **FL** | **132-223** | **61-111** | **153-249** | **FL** |
| **Del-PPGY** | **195** (6) | **117** (14) | **103** (23) | **97** (6) | **102** (25) | **116** (28) | **154** (29) | **153** (13) | **555** (84) |
| **Q322/Y324A** | **149** (34) | **27** (12) | **83** (8) | **91** (13) | **84** (27) | **87** (19) | **134** (15) | **162** (29) | **305** (40) |
| **W326A** | **430** (109) | **14** (12) | **69** (30) | **87** (11) | **28** (18) | **112** (32) | **166** (18) | **249** (4) | **319** (23) |
| **C338A** | **86** (15) | **87** (3) | **47** (13) | **80** (23) | **54** (15) | **71** (13) | **121** (15) | **79** (13) | **80** (9) |
| **L340A** | **161** (63) | **89** (33) | **68** (12) | **96** (19) | **72** (7) | **74** (17) | **95** (6) | **102** (17) | **105** (15) |
| **K341A** | **24** (4) | **163** (9) | **85** (10) | **55** (12) | **133** (46) | **66** (13) | **82** (6) | **111** (16) | **85** (17) |
| **QPSMT/SE*** | **107** (27) | **68** (23) | **118** (18) | **99** (44) | **119** (32) | **117** (6) | **89** (8) | **69** (17) | **60** (8) |
| **Y297A** | **114** (34) | **133** (14) | **69** (6) | **44** (26) | **70** (5) | **96** (31) | **77** (7) | **40** (19) | **78** (21) |
| **V224A** | **269** (130) | **321** (48) | **130** (19) | **87** (12) | **150** (44) | **76** (20) | **217** (22) | **277** (18) | **137** (34) |
| **Y226A** | **81** (10) | **766** (161) | **124** (16) | **123** (22) | **133** (38) | **100** (22) | **225** (22) | **301** (41) | **609** (22) |
| **V356R** | **4** (3) | **630** (56) | **75** (12) | **69** (31) | **113** (33) | **144** (26) | **285** (22) | **178** (33) | **199** (21) |
| **W406A** | **2** (1) | **28** (5) | **103** (11) | **130** (13) | **143** (42) | **235** (53) | **538** (45) | **714** (34) | **205** (22) |
| **D408H** | **1** (1) | **162** (16) | **111** (11) | **203** (20) | **91** (30) | **118** (19) | **72** (9) | **56** (12) | **66** (10) |

Binding between wild-type or mutant Renilla-Smad3 fusion proteins and nine different Flag epitope-tagged Smad-binding proteins was quantified by pull-down of protein complexes from cell lysates and detection of the Renilla luciferase activity per well. The Renilla luciferase counts were normalized to the number of counts recovered with phosphorylated wild-type Smad3 (100%). The proteins were all co-expressed with the constitutively active Alk5. FL indicates that the full-length protein was used. The Smad3 MH2 domain has been reported to bind directly to CBP [99], xFoxH1 [100], MyoD [60], MEF2C [61], Axin [62], Serum response factor (SRF) [91] and ATF3 [92]. The Smad3 MH1 domain mediates Smad3 binding to Hnf4 [101] and FoxO3 [93]. The amino acids comprising the CBP [31], xFoxH1 [44,100], SRF [91], ATF3 [92] and FoxO3 [93] Smad3-binding motifs expressed on the Flag-epitope tagged thioredoxin scaffold are indicated below each gene. The reduced binding to the Smad interaction motif from xFoxH1 by mutations in the alpha-helix 2 region of Smad3 (W326A and Q322A/Y324A) is consistent with previous studies mutating the corresponding amino acids in Smad2 [44]. Reduced binding to the Smad interaction motif from CBP by Smad3 W406A also is consistent with a prior study on this protein interaction that used size exclusion chromatography with purified proteins [64]. Standard deviations are indicated in parenthesis.

* The Smad3 QPSMT/SE mutant has residues 252-256 and 266-267 changed to the Smad1 equivalent amino acids (STSVL/NKN) [47].
