# Supplementary material for: Mutations in Protein-Binding Hot-Spots on the Hub Protein Smad3 Differentially Affect Its Protein Interactions and Smad3-Regulated Gene Expression
Source: PLoS One. 2011 Sep 19;6(9):e25021. doi: 10.1371/journal.pone.0025021 (PMC3176292; doi:10.1371/journal.pone.0025021)
Supplement: Table S2 — Effects of single alanine mutations in the Smad3 MH2 region implicated in binding to Ski on interactions with Smad3-binding proteins. Binding between wild-type or mutant Renilla-Smad3 fusion proteins and eight different Flag epitope-tagged Smad-binding proteins was quantified by pull-down of protein complexes from cell lysates and detection of the Renilla luciferase activity per well. The Renilla luciferase counts were normalized to the number of counts recovered with phosphorylated wild-type Smad3 (100%). The proteins were co-expressed with the constitutively active Alk5. FL indicates that the full-length protein was used. The amino acids comprising the SARA [32], Smurf2 [52], Ski [22], and Sip1 [94] Smad3-binding motifs expressed on the Flag-epitope tagged thioredoxin scaffold are indicated below each gene. The reduced binding by Smad3 with mutations in the QPSMT/SE motifs to the Ski Smad-binding domain and full length SkiW274E is consistent with previous studies [47]. The S254A mutation was the only single amino acid mutation that significantly reduced binding to the Ski Smad binding domain. Unexpectedly, E267A enhanced Smad3 interactions with several Smad-binding proteins. Standard deviations are indicated in parenthesis. (DOC) [file pone.0025021.s003.doc]

| **Smad3 Mutants** | **Percent binding to Smad3 mutants relative to the binding to wild-type Smad3 (100%)** | | | | | | | |
| --- | --- | --- | --- | --- | --- | --- | --- | --- |
|  | **Smad4** | **Smad3** | **SARA** | **SARA** | **Smurf2** | **Ski** | **SkiW274E** | **Sip1** |
|  | **FL** | **FL** | **665-721** | **FL** | **252-330** | **2-56** | **FL** | **315-487** |
| **QPSMT/SE*** | **132** (18) | **58** (4) | **61** (26) | **40** (12) | **86** (29) | **4** (1) | **32** (7) | **72** (14) |
| **Q252A** | **117** (54) | **128** (8) | **111** (8) | **190** (41) | **165** (64) | **575** (299) | **81** (13) | **120** (12) |
| **P253A** | **142** (9) | **110** (7) | **124** (10) | **218** (64) | **117** (34) | **121** (42) | **62** (24) | **100** (4) |
| **S254A** | **101** (46) | **105** (10) | **103** (32) | **154** (34) | **113** (28) | **10** (5) | **78** (25) | **128 (**103) |
| **T256A** | **183** (86) | **151** (21) | **103** (15) | **211** (27) | **137** (58) | **292** (30) | **128** (92) | **172** (77) |
| **S266A** | **81** (33) | **108** (17) | **69** (34) | **228** (37) | **92** (20) | **186** (46) | **74** (22) | **74** (37) |
| **E267A** | **458** (399) | **222** (12) | **81** (8) | **82** (20) | **379** (71) | **1152** (374) | **137** (9) | **88** (41) |

**Table S2. Effects of single alanine mutations in the Smad3 MH2 region implicated in binding to Ski on interactions with Smad3-binding proteins.**

Binding between wild-type or mutant Renilla-Smad3 fusion proteins and eight different Flag epitope-tagged Smad-binding proteins was quantified by pull-down of protein complexes from cell lysates and detection of the Renilla luciferase activity per well. The Renilla luciferase counts were normalized to the number of counts recovered with phosphorylated wild-type Smad3 (100%). The proteins were co-expressed with the constitutively active Alk5. FL indicates that the full-length protein was used. The amino acids comprising the SARA [32], Smurf2 [52], Ski [22], and Sip1 [94] Smad3-binding motifs expressed on the Flag-epitope tagged thioredoxin scaffold are indicated below each gene. The reduced binding by Smad3 with mutations in the QPSMT/SE motifs to the Ski Smad-binding domain and full length SkiW274E is consistent with previous studies [47]. The S254A mutation was the only single amino acid mutation that significantly reduced binding to the Ski Smad binding domain. Unexpectedly, E267A enhanced Smad3 interactions with several Smad-binding proteins. Standard deviations are indicated in parenthesis.
